# Methods in causal inference. Part 3: measurement error and external validity threats

**DOI:** 10.1017/ehs.2024.33

**Published:** 2024-10-01

**Authors:** Joseph A. Bulbulia

**Affiliations:** Victoria University of Wellington, New Zealand

**Keywords:** Causal inference, comparative, cross-cultural, dags, experiments, longitudinal, measurement error bias, selection bias, single world intervention graphs, SWIGs, target validity, WEIRD

## Abstract

The human sciences should seek generalisations wherever possible. For ethical and scientific reasons, it is desirable to sample more broadly than ‘Western, educated, industrialised, rich, and democratic’ (WEIRD) societies. However, restricting the target population is sometimes necessary; for example, young children should not be recruited for studies on elderly care. Under which conditions is unrestricted sampling desirable or undesirable? Here, we use causal diagrams to clarify the structural features of measurement error bias and target population restriction bias (or ‘selection restriction’), focusing on threats to valid causal inference that arise in comparative cultural research. We define any study exhibiting such biases, or confounding biases, as weird (wrongly estimated inferences owing to inappropriate restriction and distortion). We explain why statistical tests such as configural, metric and scalar invariance cannot address the structural biases of weird studies. Overall, we examine how the workflows for causal inference provide the necessary preflight checklists for ambitious, effective and safe comparative cultural research.

## Graphical abstract

**Figure:**
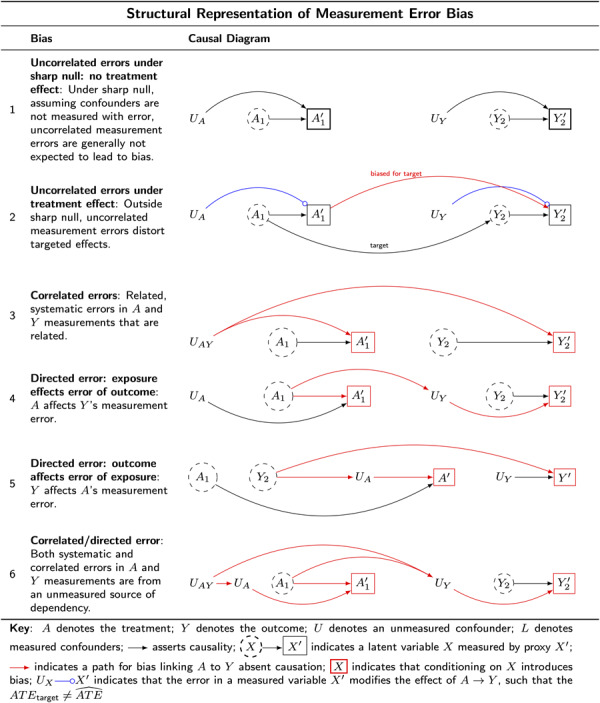

**Social media summary:** Many human scientists believe that diverse samples are essential to answer the big questions. However, advances in causal inference reveal that sampling concerns often put the cart before the horse. Investigators must first state a clear causal question and define the target population for which answers will generalise. Next, they must assess how observations can identify the causal quantities of interest, taking measurement error biases into account. Sample planning and data collection come later. This article demonstrates how these foundational principles can be precisely stated and applied to improve research design.

## Introduction

Human scientists ask and answer questions. To anchor answers in facts, we collect data.

Most publishing human scientists work in what Joseph Henrich, Steven Heine, and Ara Norenzayan have termed ‘WEIRD’ societies: ‘Western, educated, industrialised, rich, and democratic’ (Henrich et al., 2010). Unsurprisingly, WEIRD samples are over-represented in human science datasets (Arnett, 2008; Sears, 1986). Henrich et al. illustrate how WEIRD samples differ from non-WEIRD samples in areas such as spatial cognition and perceptions of fairness, while showing continuities in basic emotion recognition, positive self-views and motivation to punish anti-social behaviour. Because science seeks generalisation wherever it can, Henrich et al. urge that sampling from non-WEIRD populations is desirable.

Recently, a host of institutional diversity and inclusion initiatives have been developed that commend researchers to obtain data from global samples. In my view, the motivation for these mission statements is ethically laudable. The injunction for a broader science of humanity also accords with institutional missions. For example, the scientific mission of the American Psychological Association (APA) is ‘to promote the advancement, communication, and application of psychological science and knowledge to benefit society and improve lives’. The APA does not state that it wants to understand and benefit only North Atlantic Societies (https://www.apa.org/pubs/authors/equity-diversity-inclusion, accessed March 2024). It is therefore tempting to use such a mission statement as an ideal by which to evaluate the samples used in human scientific research.

Suppose we agree that promoting a globally diverse science makes ethical sense. Set aside the worry that global studies often do not sample the globe very well (this problem is discussed in Ghai et al., 2024). Does the sampling of globally diverse populations always advance this ideal? It is easy to find examples in which restricting our source population makes better scientific sense. Suppose we are interested in the psychological effects of restorative justice among victims of violent crime. Here, it would make little scientific sense to sample from a population that has not experienced violent crime. Nor would it make ethical sense. The scientific question, which may have important ethical implications, is not served by casting a wider net. Suppose we want to investigate the health effects of calorie restriction. It might be unethical to include children or the elderly. It makes little sense to investigate the psychological impact of vasectomy in biological females or hysterectomy in biological males.

In the cases we just considered, the scientific questions pertained to a sub-sample of the human population and so could be sensibly restricted (refer also to Gaechter, 2010; Machery, 2010). However, even for questions that relate to all of humanity, sampling from all of humanity might be undesirable. For example, if we were interested in the effects of a vaccine on disease, sampling from one population might be as good as sampling from all. Sampling from one population might spare time and expense. We might conclude that sampling universally, where unnecessary, is wasteful and unethical.

We might agree with our mission statements in judging that ethical aspirations must guide research at every phase. More fundamentally, we cannot assess the bandwidth of human diversity from the armchair, without empirical study, and this is a motivation to investigate. Yet mistaking our aspirations for sampling directives risks wasteful science. Because waste carries opportunity costs, wasteful science is unethical science.

I present these examples to remind ourselves of the importance of addressing questions of sampling in relation to the scientific question at hand.

During the past 20 years, causal data science, also known as ‘causal inference’ or ‘CI’, has enabled tremendous clarity for questions of research design and analysis (Richardson & Rotnitzky, 2014). Here, we examine how the workflows developed for causal inference clarify threats and opportunities for causal inference in comparative human research. These workflows require that we state our causal question in terms of well-defined counterfactual quantities, state the population of interest, and evaluate assumptions under which it is possible to obtain valid quantitative estimates of the counterfactual quantities we seek from data. Application of these workflows to comparative questions enables us to clarify when comparative research is possible, and also whether it is desirable. Not all questions are causal, of course. However, because manifest associations in a dataset may not be evidence of *association* in the world, even those who seek comparative descriptive understanding may benefit from causal inference workflows (Vansteelandt & Dukes, 2022).

In the remainder of the introduction, I review causal directed acyclic graphs (causal DAGs). Readers familiar with causal directed acyclic graphs may skip this section. I encourage readers unfamiliar with causal directed acyclic graphs to develop familiarity before proceeding (Barrett, 2021; Bulbulia, 2024b; Hernán & Robins, 2024: chapter 6; McElreath, 2020: chapters 5 and 6; Neal, 2020; Pearl, 2009). Because directed acyclic graphs encode causal assumptions, we will use the terms ‘structural’ and ‘causal’ synonymously.

Part 1 uses causal diagrams to clarify five structural features of measurement-error bias. Understanding measurement error bias is essential in all research, especially in comparative human science, where it casts a long shadow.

Part 2 examines structural sources of bias arising from attrition and non-response, also known as ‘right-censoring’ or simply ‘censoring’. Censoring may lead to restriction of the target population at the end of study, even if the analytic sample population at baseline represents the target population. Put differently, even if we succeed in capturing the relevant diversity of humanity when selecting cases into a study, and even if there is no confounding bias (say we have randomised treatments), we nevertheless lack assurances for valid inference at the end of study. We will explain why avoiding WEIRD samples (Western, Educated, Industrialised, Rich, Democratic) does not necessarily prevent weird (wrongly estimated inferences due to inappropriate restriction and distortion) inferences. If the analytic sample population at baseline is meant to be the target population, censoring at baseline may lead to bias.

Part 3 addresses biases that arise at the start of a study when there is a mismatch between the analytic sample population and the target population. When the target population is restricted in the analytic sample population at baseline, results may be biased. I focus on structural threats to inference when the analytic sample population is (1) too restrictive (e.g. too WEIRD – western, educated, industrialised, rich and democratic) and (2) insufficiently restrictive (leading to bias from WEIRD sampling). We find that population-restriction biases are formally equivalent to certain measurement error biases. This structural parallel is crucial because it shows that many biases in comparative research can be treated as measurement error biases. As these biases are structural – causal in nature – they cannot be assessed using the statistical estimation methods typically employed by comparative researchers.

Part 4 uses single world intervention graphs (SWIGs) to enhance understanding of measurement-error bias, which is not easily conveyed through causal DAGs. Causal DAGs are designed to evaluate assumptions of ‘no unmeasured confounding’. Consequently, they do not fully elucidate population-restriction and measurement-error biases that do not stem from confounding. Although SWIGs are also built to evaluate ‘no unmeasured confounding’, they represent counterfactual dependencies directly on a graph. By graphing the measurements – or ‘reporters’ – of latent realities we aim to quantify, along with the variables that perturb these reporters so that the reported quantities differ from the latent realities, we can advance the structural understanding of measurement problems. This approach better diagnoses threats to comparative human science and elucidates their remedies.

The importance of causal inference for comparative research has been highlighted in several recent studies (Bulbulia, 2022; Deffner et al., 2022). Here, I focus on challenges arising from structural features of (1) measurement error bias, (2) target population restriction bias from censoring and (3) target population restriction bias at a study's baseline. I clarify that the basis of these biases is causal, not statistical, by demonstrating their roots in measurement error bias. This understanding is essential because comparative researchers often rely on statistical methods, such as configural scalar and metric invariance, to address measurement issues. However, if the problems are causal, such methods are inadequate. They fail to clarify the dependencies between reality, its measurements and the contextual and cultural features that modify the effects of reality on its measurements (VanderWeele, 2022; VanderWeele & Vansteelandt, 2022).

I begin with a brief overview of causal inference, causal DAGs, and our terminology.

### What is causality?

To quantify a causal effect, we must contrast the world as it is – in principle, observable – with the world as it might have been – in principle, not observable.

Consider a binary treatment variable *A* ∈ {0, 1} representing the randomised administration of a vaccine to individuals *i* in the set {1, 2, …, *n*}. *A*_*i*_ = 1 denotes vaccine administration and *A*_*i*_ = 0 denotes no vaccine. The potential outcomes for each individual are *Y*_*i*_(0) and *Y*_*i*_(1), representing outcomes yet to be realised before administration. Thus, they are called ‘potential’ or ‘counterfactual’ outcomes. For an individual *i*, we define a causal effect as the contrast between the outcome observed under one intervention level and the outcome observed under another. This contrast, for the *i*^th^ individual, can be expressed on the difference scale as:

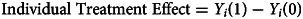

where the ‘Individual Treatment Effect’ is the difference in the outcomes for an individual under two treatment conditions, where *Y*_*i*_(1) − *Y*_*i*_(0) ≠ 0 denotes a causal effect of *A* on *Y* for individual *i* on the difference scale. Similarly,

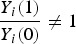

denotes a causal effect of treatment *A* for individual *i* on the risk ratio scale. These quantities cannot be computed from observational data for any individual *i*. The inability to observe individual-level causal effects is the *fundamental problem of causal inference* (Holland, 1986; Rubin, 1976). This problem has long puzzled philosophers (Hume, 1902; Lewis, 1973). However, although individual causal effects are unobservable, we may nevertheless recover average causal effects for a population, or for strata within a population, from observations. We next consider how experiments recover causal effects within populations and subpopulations.

### How we obtain average causal effect estimates from ideally conducted randomised experiments

The average treatment effect (ATE) measures the difference in outcomes between treated and control groups:
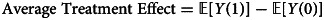
Here, 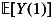 and 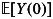 represent the average outcome for the target population if *everyone* in the population were subjected to the treatment and control conditions, respectively.

In a randomised experiment, we estimate these averages assuming that the analytic sample population matches the target population. We do this by considering the average observed and unobserved outcomes under the treatment conditions:
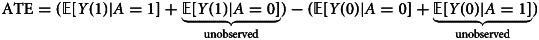
Effective randomisation ensures that potential outcomes are similarly distributed across both groups. Thus, any differences in the averages of the treatment groups can be attributed to the treatment. Therefore, in an ideally conducted randomised experiment, the average outcomes are expected to be equal across different treatment conditions for the population from which the sample is drawn:
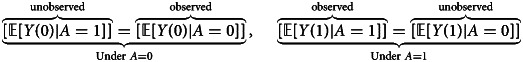
Because treatment groups are exchangeable, by randomisation, it follows that an ideally randomised controlled experiment provides an unbiased estimate of the average treatment effect:

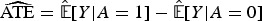

Note that in the context of our imagined experiment, 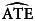 applies to the population from which the experimental participants were drawn and is calculated on the difference scale. A more explicit notation would define this effect estimate by referencing its scale and population: 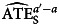, where *a*^′^ − *a* denotes the difference scale and *S* denotes the source population. I will return to this point in Parts 2 and 3, but it is important to build intuition early that in causal inference we must specify: (1) the causal effect of interest; (2) a scale of contrast; and (3) a target population for whom a causal effect estimate is meant to generalise.

### Three fundamental assumptions for causal inference

An observational study aims to estimate the average treatment effects without researchers controlling treatments or randomising treatment assignments. We can consistently estimate counterfactual contrasts only under strict assumptions. Three fundamental assumptions are required to obtain the counterfactual quantities required to compute causal contrasts from observational data.

## Assumption 1: causal consistency

Causal consistency states that the observed outcome for each individual under the treatment they actually received is equal to their potential outcome under that treatment. This means if an individual *i* received treatment *A*_*i*_ = 1, their observed outcome *Y*_*i*_ is the same as their potential outcome under treatment, denoted as *Y*_*i*_(1). Similarly, if they did not receive the treatment (*A*_*i*_ = 0), their observed outcome is the same as their potential outcome without treatment, denoted as *Y*_*i*_(0), such that:
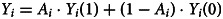
where *Y*_*i*_ denotes the observed outcome for individual *i*; *A*_*i*_ denotes the treatment status for individual *i*, with *A*_*i*_ = 1 indicating treatment received and *A*_*i*_ = 0 indicating no treatment; and *Y*_*i*_(1) and *Y*_*i*_(0) denote the potential outcomes for individual *i* under treatment and no treatment, respectively (refer to Morgan & Winship, 2014; VanderWeele, 2009).

The causal consistency assumption is necessary to link the theoretical concept of potential outcomes – the target quantities of interest – with observable data (see Bulbulia et al., 2023).

## Assumption 2: conditional exchangeability (or ignorability)

Conditional exchangeability states that given a set of measured covariates *L*, the potential outcomes are independent of the treatment assignment. Once we control for *L*, the treatment assignment *A* is as good as random with respect to the potential outcomes:
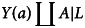
where *Y*(*a*) denotes the potential outcomes for a particular treatment level *a*; denotes conditional independence; *A* denotes the treatment levels to be contrasted; and *L* denotes the measured covariates.

Under the conditional exchangeability assumption, any differences in outcomes between treatment groups can be attributed to the treatment. This assumption requires that all confounding variables affecting both the treatment assignment *A* and the potential outcomes *Y*(*a*) are measured and included in *L*.

## Assumption 3: positivity

The positivity assumption requires that every individual in the population has a non-zero probability of receiving each treatment level, given their covariates (Bulbulia et al., 2023; Chatton et al., 2020; Hernán & Robins, 2024; Westreich & Cole, 2010). Formally,
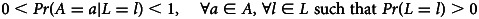
where *A* denotes the treatment or exposure variable; and *L* denotes a vector of covariates assumed sufficient for satisfying conditional exchangeability.

For a discussion of causal assumptions in relation to external validity, refer to Imai et al. (2008).

### Terminology

To avoid confusion, we define the meanings of our terms:

- *Unit/individual –* an entity, such as an object, person, or culture. We will use the term ‘individual’ instead of the more general term ‘unit’. Think ‘row’ in a dataset.
- *Variable* – a feature of an individual, transient or permanent. For example, ‘Albert was sleepy but is no longer’ or ‘Alice was born 30 November’.
- *Treatment –* equivalent to ‘exposure’, an event that might change a variable. For instance, ‘Albert was sleepy; we intervened with coffee; he is now wide awake’ or ‘Alice was born in November; nothing can change that’.
- *Outcome* – the response variable or ‘effect’. In causal inference, we contrast ‘potential’ or ‘counterfactual outcomes’. In observational or ‘real-world’ studies where treatments are not randomised, the assumptions for obtaining contrasts of counterfactual outcomes are typically much stronger than in randomised controlled experiments.
- *Confounding –* a state where the treatment and outcome share a common cause and no adjustment is made to remove the non-causal association, or where the treatment and outcome share a common effect, and adjustment is made for this common effect, or when the effect of the treatment on the outcome is mediated by a variable which is conditioned upon. In each case, the observed association will not reflect a causal association. Causal directed acyclic graphs clarify strategies for confounding control.
- *Measurement* – a recorded trace of a variable, such as a column in a dataset. When placing measurements within causal settings, we call measurements *reporters*.
- *Measurement error* – a misalignment between the true state of a variable and its reported state. For example, ‘Alice was born on 30 November; records were lost, and her birthday was recorded as 1 December’.
- *Population* – an abstraction from statistics, denoting the set of all individuals defined by certain features. Albert belongs to the set of all individuals who ignore instructions.
- *Super-population –* an abstraction, the population of all possible individuals of a given kind. Albert and Alice belong to a super-population of hominins.
- *Restricted population –* population *p* is restricted relative to another population *P* if the individuals *p* ∈ *P* share some but not all features of *P*. ‘The living’ is a restriction of hominins.
- *Target population* – a restriction of the super-population whose features interest investigators. An investigator who defines their interests is a member of the population of ‘good investigators’.
- *Source population* – the population from which the study's sample is drawn. Investigators wanted to recruit from a general population but recruited from the pool of first-year university psychology students.
- *Sample population at baseline* – or equivalently the ‘*analytical sample population*.’ The abstract set of individuals from which the units in a study at treatment assignment belong, e.g. ‘the set of all first-year university psychology students who might end up in this study’. Unless stated otherwise, we will consider the baseline analytic sample population to represent the *source population*; we will consider the *analytic population* at baseline to be representative of the *target population*.
- *Selection into the analytic sample –* selection occurs and is under investigator control when a target population is defined from a super-population or when investigators apply eligibility criteria for inclusion in the analytic sample. Selection into the sample is often out of the investigator's control. Investigators might aspire to answer questions about all of humanity but find themselves limited to undergraduate samples. Investigators might sample from a source population but recover an analytic sample that differs from it in ways they cannot measure, such as mistrust of scientists. There is typically attrition of an analytic sample over time, and this is not typically fully within investigator control. Because the term ‘selection’ has different meanings in different areas of human science, we will speak of ‘target population restriction at the start of study’. Note that to evaluate this bias, it is important for investigators to state a causal effect of interest with respect to *the full data* that include the counterfactual quantities for the treatments to be compared in a clearly defined target population where all members of the target population are exposed to each level of treatment to be contrasted (Lu et al., 2022; Westreich et al., 2017).
- *(Right) censored analytic sample at the end of study –* right censoring is generally uninformative if there is no treatment effect for everyone in the baseline population (the sharp causal null hypothesis). Censoring is informative if there is an effect of the treatment, and this effect varies in at least one stratum of the baseline population (Hernán, 2017). If no correction is applied, unbiased effect estimates for the analytic sample will bias causal effect estimates for the target population in at least one measure of effect (Greenland, 2009; Lash et al., 2020; VanderWeele, 2012). We call such bias from right censoring ‘target population restriction at the end of study’. Note again that to evaluate this bias, the causal effect of interest must be stated with respect to *the full data* that includes the counterfactual quantities for the treatments to be compared in a clearly defined target population where all members of the target population are exposed to each level of treatment to be contrasted (Westreich et al., 2017).
- *Target population restriction bias* – bias occurs when the distribution of effect modifiers in the analytic sample population differs from that in the target population, at the start, at the end, or throughout the study. Here we consider *target population restriction bias at the start of study* and *target population restriction bias at the end of study*. If this bias occurs at the start of the study, it will generally occur at the end of the study (and at intervals between), except by accident. We require validity to be non-accidental.
- *Generalisability –* a study's findings generalise to a target population if the effects observed in the analytic sample at the end of study are also valid for the target population for structurally valid reasons (i.e. non-accidentally).
- *Transportability* – when the analytic sample is not drawn from the target population, we cannot directly generalise the findings. However, we can transport the estimated causal effect from the source population to the target population under certain assumptions. This involves adjusting for differences in the distributions of effect modifiers between the two populations. The closer the source population is to the target population, the more plausible the transportability assumptions and the less we need to rely on complex adjustment methods see (Refer to supplementary materials S2).
- *Marginal effect* – typically a synonym for the average treatment effect – always relative to some population specified by investigators.
- *Intention-to-treat effect* – the marginal effect of random treatment assignment.
- *Per-protocol effect* – the effect of adherence to a randomly assigned treatment if adherence was perfect (Hernán & Robins 2017). We have no guarantee that the intention-to-treat effect will be the same as the per-protocol effect. A safe assumption is that:
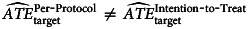

When evaluating evidence for causality, in addition to specifying their causal contrast, effect measure and target population, investigators should specify whether they are estimating an intention-to-treat or per-protocol effect (Hernán, 2004; Tripepi et al., 2007).

- *WEIRD –* a sample of ‘western, educated, industrialised, rich, and democratic societies’ (Henrich et al., 2010).
- *weird* (wrongly estimated inferences owing to inappropriate restriction and distortion) – a causal effect estimate that is not valid for the target population, either from confounding bias, measurement error bias, target population restriction at the start of study, or target population restriction at the end of study.

For discussion of these concepts refer to Dahabreh et al. (2021), Imai et al. (2008), Cole and Stuart (2010) and Westreich et al. (2017). A clear decomposition of key concepts needed to external validity – or what we call ‘target validity’ – is given in Imai et al. (2008). For a less technical, pragmatically useful discussion, refer to Stuart et al. (2018). Note that terminology differs across the causal inference literature. See supplementary materials S1 for a causal inference glossary.

### Graphical conventions

- *A* – denotes the ‘treatment’ or ‘exposure’, a random variable, ‘the cause’.
- *Y* – denotes the outcome or response, measured at the end of the study. *Y* is the ‘effect’.
- *L* – denotes a measured confounder or set of confounders.
- *U* – denotes an unmeasured confounder or confounders.
- *R* – denotes randomisation to treatment condition 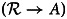.
- *Node* – denotes characteristics or features of units within a population on a causal diagram, that is, a ‘variable’. In causal directed acyclic graphs, nodes are drawn with respect to the *target population*, which is the population for whom investigators seek causal inferences (Suzuki et al., 2020). Time-indexed nodes: *X*_*t*_ denotes relative chronology.
- *Edge without an Arrow* (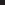) – path of association, causality not asserted.
- *Red edge without an arrow* (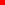) – Confounding path, ignoring arrows to clarify statistical dependencies.
- *Arrow* (→) – denotes a causal relationship from the node at the base of the arrow (a ‘parent’) to the node at the tip of the arrow (a ‘child’). In causal directed acyclic graphs, it is conventional to refrain from drawing an arrow from treatment to outcome to avoid asserting a causal path from *A* to *Y* because we aim to ascertain whether causality can be identified for this path. All other nodes and paths – including the absence of nodes and paths – are typically assumed.
- *Red arrow* (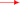) – denotes a path of non-causal association between the treatment and outcome. Despite the arrows, this path is associational and may flow against time.
- *Open blue arrow* (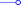) – denotes effect modification, which occurs when the effect of treatment varies within levels of a covariate. We do not assess the causal effect of the effect modifier on the outcome, recognising that it may be incoherent to consider intervening on the effect modifier. However, if the distribution of effect modifiers in the analytic sample population differs from that in the target population, then at least one measure of causal effect will differ between the two populations.
- *Boxed variable* (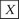)– denotes conditioning or adjustment for *X*.
- *Red-boxed variable* (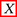) – highlights the source of confounding bias from adjustment.
- *Dashed circle* (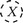) – denotes no adjustment is made for a variable (implied for unmeasured confounders).
- *G –* names a causal diagram.
- *Split node (SWIGs)* (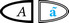) – convention used in single world intervention graphs (SWIGs) that allows for factorisation of counterfactuals by splitting a node at an intervention with post-intervention nodes relabelled to match the treatment. We introduce single world intervention graphs in Part 4.
- *Unobserved node (SWIGs)* (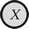) – our convention when using single world intervention graphs to denote an unobserved node (SWIGs): *X* unmeasured.

### Causal directed acyclic graphs (DAGs)

Judea Pearl proved that, based on assumptions about causal structure, researchers can identify causal effects from joint distributions of observed data (Pearl, 1995, 2009). The rules of d-separation are given in Table 1.

**Table 1.** Five elementary causal structures in a causal directed acyclic graph

Five Elementary Causal Structures				
Structure		Causal DAG	Explanation	Implication
**Two variables**				
1.	**Causality Absent**	A B	*A* and *B* have no causal effect on each other	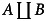
2.	**Causality Present**	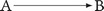	*A* causally affects *B*, and they are associated	*A* 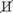 *B*
**Three variables**				
3.	**Fork**	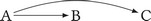	*A* causally affects both *B* and *C*; *B* and *C* are conditionally independent given *A*	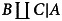
4.	**Chain**	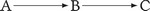	*C* is affected by *B* which is, in turn, affected by *A*; *A* and *C* are conditionally independent given *B*	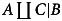
5.	**Collider**	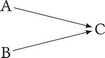	*C* is affected by both *A* and *B*, which are independent; conditioning on *C* induces association between *A* and *B*	*A* 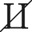 *B*|*C*

**Key**: 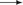, a directed edge, denotes causal association. The absence of an arrow denotes no causal association. **Rules of d-separation**: In a causal diagram, a path is ‘blocked’ or ‘d-separated’ if a node along it interrupts causation. Two variables are d-separated if all paths connecting them are blocked or if there are no paths linking them, making them conditionally independent. Conversely, unblocked paths result in ‘d-connected’ variables, implying statistical association. Refer to Pearl (1995).

Note that ‘d’ stands for ‘directional’.

Implication: 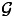 denotes a causal directed acyclic graph (causal DAG). P denotes a probability distribution function. Pearl proved that independence in a causal DAG 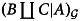 implies probabilistic independence 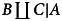)*_P_*; likewise if (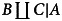)*_P_* holds in all distributions compatible with 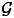, it follows that (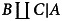)*_G_* (refer to Pearl 2009, p.61.) We read causal graphs to understand the implications of causality for relationships in observable data. However, reading causal structures from data is more challenging because the relationships in observable data are typically compatible with more than one (and typically many) causal graphs.

Pearl's rules of d-separation can be stated as follows:

- *Fork rule* (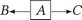) –*B* and *C* are independent when conditioned on *A* (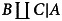).
- *Chain rule* (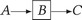) – conditioning on *B* blocks the path between *A* and *C* (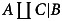).
- *Collider rule* (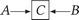)–*A* and *B* are independent until conditioned on *C*, which introduces dependence 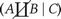.

Table 1 shows causal directed acyclic graphs corresponding to these rules. Because all causal relationships can be assembled from combinations of the five structures presented in Table 1, we can use causal graphs to evaluate whether and how causal effects may be identified from data (Bulbulia, 2024b).

Pearl's general identification algorithm is known as the ‘back door adjustment theorem’ (Pearl, 2009).

#### Backdoor adjustment

In a causal DAG, a set of variables *L* satisfies the backdoor adjustment theorem relative to the treatment *A* and the outcome *Y* if *L* blocks every path between *A* and *Y* that contains an arrow pointing into *A* (a backdoor path). Formally, *L* must satisfy two conditions:

1. *No path condition –* no element of *L* is a descendant of *A*.
2. *Blocking condition – L* blocks all backdoor paths from *A* to *Y*.

If *L* satisfies these conditions, the causal effect of *A* on *Y* can be estimated by conditioning on 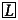 (Pearl, 2009).

## Effect-modification on causal directed acyclic graphs

The primary function of a causal directed acyclic graph is to allow investigators to apply Pearl's backdoor adjustment theorem to evaluate whether causal effects may be identified from data, as shown in Table 1. We have noted that modifying a causal effect within one or more strata of the target population opens the possibility for biased average treatment effect estimates when the distribution of these effect modifiers differs in the analytic sample population (Bulbulia, 2024c).

We do not generally represent non-linearities in causal directed acyclic graphs, which are tools for obtaining relationships of conditional and unconditional independence from assumed structural relationships encoded in a causal diagram that may lead to a non-causal treatment/outcome association (Bulbulia, 2024b).

Table 2 presents our convention for highlighting a relationship of effect modification in settings where (1) we assume no confounding of treatment and outcome and (2) there is effect modification such that the effect of *A* on *Y* differs in at least one stratum of the target population.

**Table 2.** The five elementary structures of causality which all directed acyclic graphs are composed

Conventions for Effect Modification: We assume 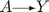		
Symbol	Meaning	Example
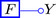	**Boxed blue variable and blue path:** observed effect-modification. **Blue arrow need not have a causal interpretation**	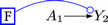
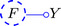	**Dashed blue circle and blue path:** effect-modifier not conditioned upon. **Blue arrow need not have a causal interpretation**	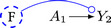

To focus on effect modification, we do not draw a causal arrow from the direct effect modifier *F* to the outcome *Y*. This convention is specific to this article (refer to Hernán & Robins, 2024: 126–127, for a discussion of ‘non-causal’ arrows).

## Part 1: how measurement error bias makes your causal inferences weird (wrongly estimated inferences owing to inappropriate restriction and distortion)

Measurements record reality, but they are not always accurate. Whenever variables are measured with error, our results can be misleading. Every study must therefore consider how its measurements might mislead.

Causal graphs can deepen understanding because – as implied by the concept of ‘record’ – there are structural or causal properties that give rise to measurement error. Measurement error can take various forms, each with distinct implications for causal inference:

- *Independent (undirected)/uncorrelated* – errors in different variables do not influence each other.
- *Independent (undirected) and correlated* – errors in different variables are related through a shared cause.
- *Dependent (directed) and uncorrelated* – errors in one variable influence the measurement of another, but these influences are not related through a shared cause.
- *Dependent (directed) and correlated* – errors in one variable influence the measurement of another, and these influences are related through a shared cause (Hernán & Cole, 2009; VanderWeele & Hernán, 2012).

The six causal diagrams presented in Table 3 illustrate structural features of measurement error bias and clarify how these structural features compromise causal inferences.

**Table 3.** Examples of measurement error bias

Structural Representation of Measurement Error Bias		
Bias		Causal Diagram
1	**Uncorrelated errors under sharp null: no treatment effect**: Under sharp null, assuming confounders are not measured with error, uncorrelated measurement errors are generally not expected to lead to bias	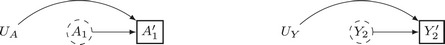
2	**Uncorrelated errors under treatment effect**: Outside sharp null, uncorrelated measurement errors distort targeted effects	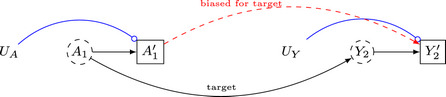
3	**Correlated errors**: Related, systematic errors in A and Y measurements that are related	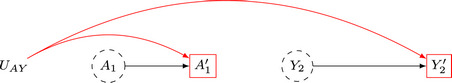
4	**Directed error: exposure effects error of outcome**: A affects Y's measurement error	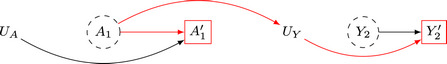
5	**Directed error: outcome affects error of exposure**: Y affects A's measurement error	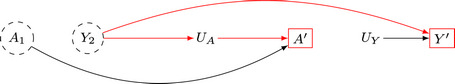
6	**Correlated/directed error**: Both systematic and correlated errors in A and Y measurements are from an unmeasured source of dependency	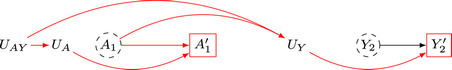

**Key**: *A* denotes the treatment; *Y* denotes the outcome; *U* denotes an unmeasured confounder; *L* denotes measured confounders; 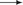 asserts causality; 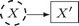 indicates a latent variable *X* measured by proxy *X′*; 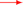 indicates a path for bias linking *A* to *Y* absent causation; 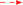 biased path for treatment effect in the target population; 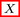 indicates that conditioning on *X* introduces bias; 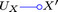 indicates that the error in a measured variable *X′* modifies the effect of *A* → *Y*, such that the 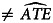.

Understanding the structural features of measurement error bias will help us understand why measurement error bias cannot typically be evaluated with statistical models and will prepare us to understand how target-population restriction biases are linked to measurement error.

### Example 1: uncorrelated non-differential errors under sharp null – no treatment effect

Table 3 *G*_1_ illustrates uncorrelated non-differential measurement error under the ‘sharp-null,’ which arises when the error terms in the exposure and outcome are independent. In this setting, the measurement error structure is not expected to produce bias.

For example, consider a study investigating the causal effect of beliefs in big Gods on social complexity in ancient societies. Imagine that societies either randomly omitted or inaccurately recorded details about their beliefs in big Gods and their social complexities. This might occur because of varying preservation in the records of cultures which is unrelated to the actual beliefs or social complexity. In this scenario, we imagine the errors in historical records for beliefs in big Gods and for social complexity are independent. When the treatment is randomised, uncorrelated and undirected errors will generally not introduce bias *under the sharp null of no treatment effect for any unit* when all backdoor paths are closed. However, if confounders are measured without error this may open a backdoor path from treatment to outcome. For example, Robins and Hernán (2008: 2216) discuss how in non-experimental settings, mismeasured confounders can introduce bias even when the measurement errors of the treatment and outcome are uncorrelated and undirected and there is no treatment effect. This is because mismeasured confounders will not control for confounding bias. We present an illustration of this bias in Table 6, G_3 where we discuss challenges to comparative research in which the accuracy of confounder measurements varies across the sites to be compared.

### Example 2: uncorrelated non-differential errors ‘off the null’ (true treatment effect) biases true effects towards the null

Table 3 *G*_2_ illustrates uncorrelated non-differential measurement error, which arises when the error terms in the exposure and outcome are independent This bias is also called information bias (Lash et al., 2009). In this setting, bias will often attenuate a true treatment effect. However, there are no guarantees that uncorrelated undirected measurement error biases effect estimates towards the null (Jurek et al., 2005, 2006, 2008; Lash et al., 2009: 93).

Consider again the example of a study investigating a causal effect of beliefs in big Gods on social complexity in ancient societies, where there are uncorrelated errors in the treatment and outcome. In this case, measurement error will often make it seem that the true causal effects of beliefs in big Gods are smaller than they are, or perhaps even that such an effect is absent. Often but not always: again, attenuation of the effect estimate is not guaranteed, and mismeasured confounders will open backdoor paths. We can, however, say this: uncorrelated undirected measurement error in the presence of a true effect leads to distortion of that effect, inviting weird results (wrongly estimated inferences owing to inappropriate restriction and distortion).

### Example 3: correlated errors non-differential (undirected) measurement errors

Table 3 *G*_3_ illustrates the structure of correlated non-differential (undirected) measurement error bias, which arises when the error terms of the treatment and outcome share a common cause.

Consider an example: imagine that societies with more sophisticated record-keeping systems tend to offer more precise and comprehensive records of both beliefs in big Gods and of social complexity. In this setting, it is the record-keeping systems that give the illusion of a relationship between big Gods and social complexity. This might occur without any causal effect of big-God beliefs on measuring social complexity or vice versa. Nevertheless, the correlated sources of error for both the exposure and outcome might suggest causation in its absence.

Correlated non-differential measurement error invites weird results (wrongly estimated inferences owing to inappropriate restriction and distortion).

### Example 4: uncorrelated differential measurement error: exposure affects error of outcome

Table 3 *G*_4_ illustrates the structure of uncorrelated differential (or directed) measurement error, where a non-causal path is opened linking the treatment, the outcome or a common cause of the treatment and outcome.

Continuing with our previous example, imagine that beliefs in big Gods lead to inflated records of social complexity in a culture's record-keeping. This might happen because the record keepers in societies that believe in big Gods prefer societies to reflect the grandeur of their big Gods. Suppose further that cultures lacking beliefs in big Gods prefer Bacchanalian-style feasting to record-keeping. In this scenario, societies with record keepers who believe in big Gods would appear to have more social complexity than equally complex societies without such record keepers.

Uncorrelated directed measurement error bias also invites weird results (wrongly estimated inferences owing to inappropriate restriction and distortion).

### Example 5: uncorrelated differential measurement error: outcome affects error of exposure

Table 3 *G*_5_ illustrates the structure of uncorrelated differential (or directed) measurement error, this time when the outcome affects the recording of the treatment that preceded the outcome.

Suppose that ‘history is written by the victors’. Can we give a structural account of measurement error bias arising from such selective retention of the past? Suppose that social complexity causes beliefs in big Gods. Perhaps kings create big Gods after the image of kings. If the kings prefer a history in which big Gods were historically present, this might bias the historical record, opening a path of association that reverses the order of causation. Such results would be weird (wrongly estimated inferences owing to inappropriate restriction and distortion).

### Example 6: uncorrelated differential error: outcome affects error of exposure

Table 3 *G*_6_ illustrates the structure of correlated differential (directed) measurement error, which occurs when the exposure affects levels of already correlated error terms.

Suppose social complexity produces a flattering class of religious elites who produce vainglorious depictions of kings and their dominions and also of the extent and scope of their society's beliefs in big Gods. For example, such elites might downplay widespread cultural practices of worshipping lesser gods, inflate population estimates and overstate the range of the king's political economy. In this scenario, the errors of the exposure and of the outcome are both correlated and differential.

Results based on such measures might be weird (wrongly estimated inferences owing to inappropriate restriction and distortion).

### Summary

In Part 1, we examined four types of measurement error bias: independent, correlated, dependent and correlated dependent. The structural features of measurement error bias clarify how measurement errors threaten causal inferences. Considerably more could be said about this topic. For example, VanderWeele and Hernán (2012) demonstrate that, under specific conditions, we can infer the direction of a causal effect from observed associations. Specifically, if:

1. the association between the measured variables 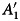 and 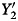 is positive;
2. the measurement errors for these variables are not correlated; and
3. we assume distributional monotonicity for the effect of *A* on *Y* (applicable when both are binary);

then a positive observed association implies a positive causal effect from *A* to *Y*. Conversely, a negative observed association provides stronger evidence for a negative causal effect if the error terms are positively correlated than if they are independent. This conclusion relies on the assumption of distributional monotonicity for the effect of *A* on *Y*. For now, the four elementary structures of measurement error bias will enable us to clarify the connections between the structures of measurement error bias, target population restriction bias at the end of a study, and target restriction bias at the start of a study.

We will return to measurement error again in Part 4. Next, we focus on structural features of bias when there is an inappropriate restriction of the target population in the analytic sample at the end of study.

## Part 2: how target population restriction bias at the end of study makes your causal inferences weird (wrongly estimated inferences owing to inappropriate restriction and distortion)

Suppose the analytic sample population at the start of a study matches the source population from which it is drawn and that this source population aligns with the target population. In this setting, at the start of study, if all goes well, there is hope that our results may generalise to the target population. Right-censoring, typically abbreviated to ‘censoring’ and also known as ‘attrition and non-response’, may bias causal effect estimates, spoiling our hopes for valid causal inferences, in one of two ways: by opening pathways of bias (distortion) or by inappropriately restricting the analytic sample population at the end of a study so that it is no longer representative of the target population. Both forms of bias will make causal inferences weird (wrongly estimated inferences owing to inappropriate restriction and distortion).

### Example 1: confounding by common cause of treatment and attrition

Table 4 *G*_1_ illustrates confounding by common cause of treatment and outcome in the censored such that the potential outcomes of the population at baseline *Y*(*a*) may differ from those of the censored population at the end of study *Y*^′^(*a*) such that *Y*^′^(*a*) ≠ *Y*(*a*).

**Table 4.** Five examples of right-censoring bias

Structural Representation of Biases from Right-Censoring		
Bias		Description
1	Confounding by common cause of treatment and attrition	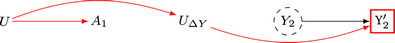
2	Treatment affects censoring	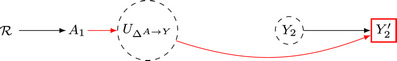
3	No treatment effect when outcome causing censoring: Bias is not expected under the strict null	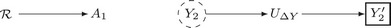
4	Treatment effect when outcome causes censoring and there is a true treatment effect. Expect a biased effect estimate for the target population on at least one causal contrast scale	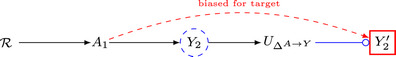
5	Treatment effect and effect-modifiers differ in censored (restriction bias without confounding)	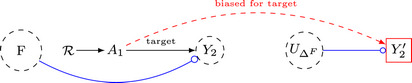

**Key**:

*A* denotes the treatment;

*Y* denotes the outcome;

*U* denotes an unmeasured confounder;

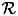
 denotes randomisation into treatment;

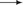
 asserts causality

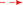
 biased path for treatment effect in the target population.

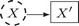
 indicates a latent variable *X* measured by proxy *X*′.

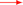
 indicates a path for bias linking *A* to *Y* absent causation.

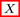
 indicates that conditioning on *X* introduces bias.

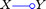
 indicates effect modification of 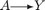 by *X*.

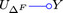
 indicates effect modification of 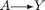 by 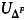

Suppose investigators are interested in whether religious service attendance affects volunteering. Suppose that an unmeasured variable, loyalty, affects religious service attendance, attrition and volunteering. The structure of this bias reveals an open backdoor path from the treatment to the outcome.

We have encountered this bias before. The structure we observe here is one of correlated measurement errors (Table 3 *G*_3_). In this example, attrition may exacerbate measurement error bias by opening a path from 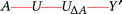


The results obtained from such a study would be distorted – that is, weird (wrongly estimated inferences owing to inappropriate restriction and distortion). Here, distortion operates through the restriction of the target population in the analytic sample population at the end of the study.

### Example 2: treatment affects censoring

Table 4 *G*_2_ illustrates bias in which the treatment affects the censoring process. Here, the treatment causally affects the outcome reporter but does not affect the outcome itself.

Consider a study investigating the effects of mediation on well-being. Suppose there is no treatment effect but that Buddha-like detachment increases attrition. Suppose those with lower Buddha-like detachment report well-being differently than those with higher Buddha-like detachment. Buddha-like detachment is not a cause of well-being, we suppose; however, we also suppose that it is a cause of measurement error in the reporting of well-being. In this setting, we discover a biasing path that runs: (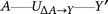). Note that there is no confounding bias here because there is no common cause of the treatment and the outcome.

We have encountered this structural bias before. The structure we observe here is one of directed uncorrelated measurement error (Table 3 *G*_4_). Randomisation ensures no backdoor paths. However, if the intervention affects both attrition and how the outcome is reported the treatment will cause measurement error bias (note this is not confounding bias because the treatment and outcome do not share a common cause.)

The results obtained from such a study risk distortation, inviting weirdness (wrongly estimated inferences owing to inappropriate restriction and distortion). Here, distortion operates through the restriction of the target population at the end of the study, assuming the analytic sample at the start of the study represented that target population (or was weighted to represent it).

### Example 3: no treatment effect when outcome causing censoring

Table 4 *G*_3_ illustrates the structure of bias when there is no treatment effect yet the outcome affects censoring.

If *G*_3_ faithfully represents reality, under the sharp null we would generally not expect bias in the average treatment effect estimate from attrition. The structure we observe here is again familiar: it is one of undirected uncorrelated measurement error (Table 3 *G*_1_). However, as before, at the start of study it is generally unclear whether the sharp null holds (if it were clear, there would be no motivation for the study). In theory, however, although the analytic sample population in the setting we have imagined would be a restriction of the target population, such a restriction of the target population is not expected to bias the null result. Again, we consider this example for its theoretical interest; no statistical test could validate what amounts to a structural assumption of the sharp null.

### Example 4: treatment effect when outcome causes censoring and there is a true treatment effect

Table 4 *G*_4_ illustrates the structure of bias when the outcome affects censoring in the presence of a treatment effect. If the true outcome is an effect modifier of the measured outcome, we can expect bias in at least one measure of effect (e.g. the risk ratio or the causal difference scale). We return to this form of bias with a worked example in Part 4, where we clarify how such bias may arise even without confounding. We shall see that the bias described in Table 4 *G*_4_ is equivalent to measurement error bias. For now, we note that the results of the study we have imagined here would be weird (wrongly estimated inferences owing to inappropriate restriction and distortion).

### Example 5: treatment effect and effect-modifiers differ in censored (restriction bias without confounding)

Table 4 *G*_5_ represents a setting in which there is a true treatment effect, but the distribution of effect-modifiers – variables that interact with the treatment – differs among the sample at baseline and the sample at the end of the study. Knowing nothing else, we might expect this setting to be standard. Where measured variables are sufficient to predict attrition, that is, where missingness is at random, we can obtain valid estimates for a treatment effect by inverse probability of treatment weighting (Cole & Hernán, 2008; Leyrat et al., 2021) or by multiple imputation – on the assumption that our models are correctly specified (Shiba & Kawahara, 2021). However, if missingness is not completely at random, or if our models are otherwise misspecified, then causal estimation is compromised (Malinsky et al., 2022; Tchetgen Tchetgen & Wirth, 2017).

Note that Table 4 *G*_5_ closely resembles a measurement structure we have considered before, in Part 1: Table 3 *G*_2_. Replacing the unmeasured effect modifiers 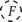 and *U*_Δ*F*_ in Table 4 *G*_5_ for 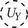 in Table 3 *G*_2_ reveals that the unmeasured effect modification in the present setting can be viewed as an example of uncorrelated independent measurement error when there is a treatment effect (i.e. censoring ‘off the null’.)

In the setting we describe in Table 4 *G*_5_ there is a common cause of the treatment and outcome. Nevertheless, the analytic sample population at the end-of-study is an undesirable restriction of the target population because the marginal effect estimate for this analytic sample population will differ from that of the target population (refer to supplementary materials S4 for a simulation that covers applies to this setting). We infer that results in this setting just described permit weirdness (wrongly estimated inferences owing to inappropriate restriction and distortion) because censoring leads to inappropriate restriction.

### Summary

In this section, we examined how right-censoring, or attrition, can lead to biased causal effect estimates. Even without confounding bias, wherever the distribution of variables that modify treatment effects differs between the analytic sample population at the start and end of the study, the average treatment effects may differ, leading to biased estimates for the target population. To address such bias, investigators must ensure that the distribution of potential outcomes at the end of the study corresponds with that of the target population. Again, methods such as inverse probability weighting and multiple imputation can help mitigate this bias (refer to Bulbulia, 2024a).

The take-home message is this: attrition is nearly inevitable, and if attrition cannot be checked it will make results weird (wrongly estimated inferences owing to inappropriate restriction and distortion). Refer to supplementary materials S3 for a mathematical explanation of why effects differ when the distribution of effect modifiers differs. Refer to supplementary materials S4 for a data simulation that makes the same point.

Next, we investigate target population restriction bias at the start of the study (left-censoring). We shall discover that structural motifs of measurement error bias reappear.

## Part 3: how target population restriction bias at the start of study makes your causal inferences weird (wrongly estimated inferences owing to inappropriate restriction and distortion)

Consider target-restriction bias that occurs at the start of a study. There are several failure modes. For example, the source population from which participants are recruited might not align with the target population. Moreover, even where there is such alignment, the participants recruited into a study – the analytic sample – might not align with the source population. For simplicity, we imagine the analytic sample population at the start of the study accurately aligns with the source population. What constitutes ‘alignment’? We say the sample is unrestrictive of the target population if there are no differences between the sample and target population in the distribution both of confounders (common causes of treatment and outcome) and of the variables that modify treatment effects (effect modifiers). Proof of alignment cannot be verified with data (refer to supplementary materials S3).

### Target population restriction bias at baseline can be collider-restriction bias

Table 5 *G*_1_ illustrates an example of target population restriction bias at baseline in which there is collider-restriction bias.

**Table 5.** Collider-Stratification bias at the start of a study (‘M-bias’)

Selection Restriction Before Start of Study Arising from Collider Stratification Bias		
Bias		Causal Graph
1	**Problem:** Sample selection may induce M-bias (M-bias): **threat to external validity**	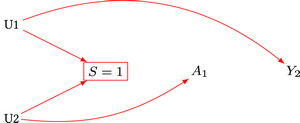
2	**Response:** (a) Do not restrict sample; (b) condition on descendent of *U*1 or *U*2	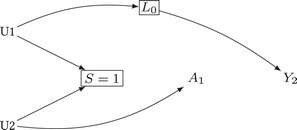

Suppose investigators want to estimate the causal effects of regular physical activity, *A*, and heart health, *Y*, among adults visiting a network of community health centres for routine check-ups.

Suppose there are two unmeasured variables that affect selection into the study *S* = 1:

1. Health awareness, *U*1, an unmeasured variable that influences both the probability of participating in the study, 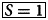, and the probability of being physically active, *A*. Perhaps people with higher health awareness are more likely to (1) engage in physical activity and (2) participate in health-related studies.
2. Socioeconomic status (SES), *U*2, an unmeasured variable that influences both the probability of participating in the study, 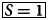, and heart health, *Y*. We assume that individuals with higher SES have better access to healthcare and are more likely to participate in health surveys; they also tend to have better heart health from healthy lifestyles: joining expensive gyms, juicing, long vacations and the like.

As presented in Table 5 *G*_1_, there is collider-restriction bias from conditioning on *S* = 1:

1. *U*1 – because individuals with higher health awareness are more likely to be both physically active and participate in the study, the subsample over-represents physically active individuals. This overestimates the prevalence of physical activity, setting up a bias in overstating the potential benefits of physical activity on heart health in the general population.
2. *U*2 – because individuals with higher SES may have better heart health from SES-related factors, this opens a confounding path from physical activity and heart health through the selected sample, setting up the investigators for the potentially erroneous inference that physical activity has a greater positive impact on heart health than it actually does in the general population. The actual effect of physical activity on heart health in the general population might be less pronounced than observed.

It might seem that researchers would need to sample from the target population. However, Table 5 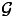 makes it clear that by adjusting for health awareness or SES or a proxy for either, researchers may block the open path arising from collider stratification bias. After such conditioning, we should expect a null effect in the sample population just as in the target population.

The next series of examples illustrates challenges to obtaining valid causal effect estimates in the presence of interactions.

### Target population restriction bias at baseline without collider-restriction bias at baseline

#### Problem 1: the target population is not WEIRD (western, educated, industrialised, rich and democratic); the analytic sample population is WEIRD

Table 6 *G*_1.1_ presents a scenario for target population restriction bias at baseline. When the analytic sample population obtained at baseline differs from the target population in the distributions of variables that modify treatment effects, effect estimates may be biased, even without confounding bias. Results may be *weird* without arising from confounding bias. This problem has been recently considered in Schimmelpfennig et al. (2024).

**Table 6.** The association in the population of selected individuals differs from the causal association in the target population. Hernán (2017) calls this scenario ‘selection bias off the null’. Lu et al. (2022) call this scenario ‘Type 2 selection bias’. We call this bias ‘target population restriction bias at baseline’

Target Population Restriction Before Start of Study		
Bias		Causal Graph
1	**Problem:** Target population is not WEIRD; sample population is WEIRD	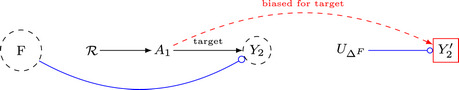
	**Response:** Sample from the target population (with assumptions)	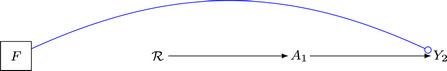
2	**Problem:** Target population is WEIRD; sample population is not WEIRD	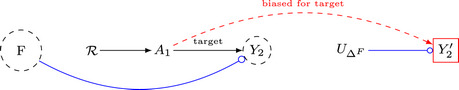
	**Response:** Eligibility restrictions	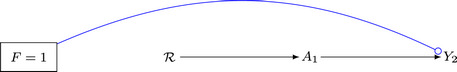
3	**Problem:** Correlated measurement error of treatment and outcome in an overly ambitious target population	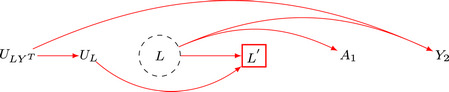
	**Response:** Eligibility restrictions for a less ambitious target	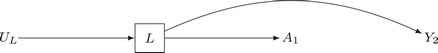
4	**Problem:** Correlated measurement error of measured effect-modifiers for an overly ambitious target population	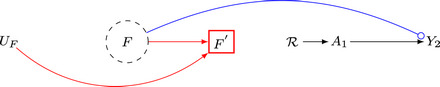
	**Response:** Eligibility restrictions for a less ambitious target population	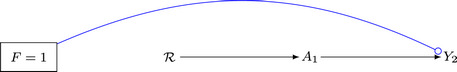

**Key**: *A* denotes the treatment; *Y* denotes the outcome; 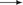 asserts causality; 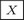 indicates conditioning on variable *X*; 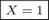 indicates fixing co-variate to level *X* = 1; 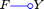 indicates effect modification of 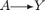 by *F*; 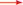 biased path for treatment effect arising from confounding by a common cause; 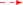 biased path for treatment effect in target population; 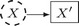 indicates a latent variable *X* measured by proxy *X*′; 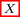 indicates that conditioning on *X* introduces bias.

Suppose we are interested in the effects of political campaigning but only sample from our preferred political party. Results for the general population will be distorted if the distribution of effect modifiers of the treatment varies by party. One such effect modifier might be ‘party affiliation’. This valid concern underscores the call for broader sampling in the human sciences. WEIRD samples will not be informative for science generally whenever the distribution of effect modifiers among humans differs from those of the restricted population of humans from which WEIRD analytic samples are drawn.

Note that we have encountered Table 6 *G*_1.1_ *twice* before. It is the same causal directed acyclic graph as we found in Table 5 *G*_5_. As we did before, we may replace the unmeasured effect modifiers 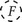 and *U*_Δ*F*_ for 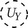 in Table 3 *G*_2_ and observe that we recover uncorrelated measurement error ‘off the null’ (i.e. when there is a true treatment effect).

The structural similarity suggests options might be easily overlooked. Where the distributions of treatment-effect modifiers are known and measured and where census (or other) weights are available for the distributions of effect modifiers in the target population, it may be possible to weight the sample to more closely approximate the target population parameters of interest (Stuart et al., 2015).

Let 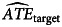 denote the population average treatment effect for the target population. Let 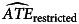 denote the average treatment effect at the end of treatment. Let *W* denote a set of variables upon which the restricted and target populations structurally differ. We say that results *generalise* if we can ensure that:
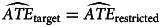
or if there is a known function such that

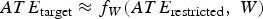

In most cases, *f*_*W*_ will be unknown, as it must account for potential heterogeneity of effects and unobserved sources of bias. For further discussion on this topic, see Imai et al. (2008), Cole and Stuart (2010) and Stuart et al. (2018).

Table 6 *G*_1.2_ provides a graphical representation of the solution.

Importantly, if there is considerable heterogeneity across humans, *then we might not know how to interpret the average treatment effect for the target population of all humans even if this causal effect can be estimated.* In comparative research, we are often precisely interested in treatment heterogeneity. If we seek explicitly comparative models, however, we will need to ensure the validity of estimates for every sample that we compare. If one stratum in the comparative study is weird (wrongly estimated inferences owing to inappropriate restriction and distortion), errors will propagate to the remainder of the comparative study. To understand such propogation consider scenarios where the target population is a deliberate restriction of the the source population from which the analytic sample at baseline is drawn. We deliberately seek restriction wherever ‘eligibility criteria’ are desirable for a study. Although this point is perhaps obvious, Although this point is perhaps obvious, many observational studies do not report eligibility criteria.

#### Example 2: the target population is a sub-sample of WEIRD (western, educated, industrialised, rich and democratic); the analytic sample population is not WEIRD enough.

Table 6 *G*_2.1_ presents a scenario where the source population does not meet eligibility criteria. Consider again the question of whether vasectomy affects a sense of meaning and purpose in life. Suppose further we want to evaluate effects in New Zealand among men over the age of 40 who have no prior history of vasectomy, and who are in relationships with heterosexual partners. The target population is a stratum of WEIRD population (western, educated, industrialised, rich and democratic). That is, the WIERD population would be too broad for scientific interest. We should not sample from young children, the elderly or any who do not qualify. Not only is it clear that a narrow population is desirable for many scientific questions, but also it is easy to imagine settings in comparative human science for which a fully unrestricted human population would be undesirable. In causal inference, we attempt to emulate ideal (although typically implausible) experiments with ‘real world’ data. Just as eligibility criteria are often useful for isolating populations of interest in experimental designs, so too are eligibility criteria often useful for isolating populations of interest in real-world ‘target trials’ (Hernán et al., 2008).

Note again Table 6 *G*_2.1_ is identical to Table 4 *G*_5_ – right-censoring bias with effect modifiers in an otherwise unconfounded study. The structure is also similar to Table 3 *G*_2_: the problem is structurally that of uncorrelated measurement error ‘off the null’. Where it is the defusion of the effect-modifiers that causes we may fix the measurement error by restricting the sample.

Table 6 *G*_2.2_ presents a solution. Ensure eligibility criteria are scientifically relevant and feasible. Sample from this eligible population. With caution, apply survey or other weights where these weights enable a closer approximation to the distributions of effect-modifiers in the target population. Notice that here we avoid weird inferences (wrongly estimated inferences owing to inappropriate restriction and distortion) by imposing greater restriction on what would otherwise be an inappropriately *unrestricted* target population.

#### Example 3: correlated measurement error of covariates and outcome in the absence of a treatment effect

Table 6 *G*_3.1_ considers the threats to external validity from correlated measurement errors in the target population arising from structured errors across heterogeneous strata. For simplicity imagine the groups with structured errors are cultures. Even if the treatment is measured without error, multiple sources of error may led to statitical association without causation.

Suppose we plan a cross-cultural investigation to clarify the relationship between interventions on religious service attendance, *A*, and charitable giving, *Y*. We plan to obtain measures of covariates *L* sufficient to control for confounding. Suppose we observe religious attendance so that it is not measured with error (as did Shaver et al., 2021), yet there is heterogeneity in the measurement of covariates *L* and the outcome *Y*. For example, if charitable giving measures are included among the baseline covariates in *L*, measurement errors at baseline will be correlated with outcome measures. Perhaps in certain cultures, charitable giving is under-reported (perhaps charity is associated with the vice of gullibility), while in others, it is over-reported (perhaps only the charitable are hired and promoted). Suppose further that true covariates affect the treatment and outcome. As shown in Table 6 *G*_3.1_, in this setting, multiple paths of confounding bias are open.

Moreover, because measurements are causally related to the phenomena we record, we cannot apply statistical tests to verify whether measures are recorded with error (VanderWeele, 2022; Vansteelandt & Dukes, 2022). Whether the phenomena that we hope to measure are functionally equivalent across cultural settings remains unknown, and can generally only be discovered slowly, through patient, careful work with local experts. Although big cross-cultural projects are preferred in certain science journals, including multiple cultures in a single analysis imposes considerable burdens on investigators. All sources of error must be evaluated – and errors from one culture can poison the wells in the analysis of others.

Table 6 *G*_3.2_ provides a sensible solution: restrict one's study to those cultures where causality can be identified. Democritus wrote, ‘I would rather discover one cause than gain the kingdom of Persia’ (Freeman, 1948). Paraphrasing Democritus, we might say, ‘I should rather discover one WEIRD cause than the kingdom of weird comparative research’.

#### Example 4: correlated measurement error of effect modifiers for an overly ambitious target population

Table 6 *G*_4.1_ considers the threats to target validity from correlated measurement errors in the target population arising from structured errors linking measurements for the effect modifiers. Here, we discover a familiar structural bias of correlated measurement error bias Table 3 *G*_3_.

Even if the treatment is randomised so that there are no open backdoor paths, and even if the treatment and outcome are measured without error, we will not be able to obtain valid estimates for treatment-effect heterogeneity from their data, nor will we be able to apply target-sample weights (such as census weights) to obtain valid estimates for the populations in which the measurement errors of effect modifiers are manifest.

Table 6 *G*_4.2_ suggests that where measures of effect modification are uncertain, it is best to consider settings in which the measurements are reliable – whether or not the settings are WEIRD (western, educated, industrialised, rich and democratic). Moreover, in comparative settings where multiple cultures are measured, unless each is proven innocent of structural measurement error bias, it is generally best to report the results for each culture separately, without attempting comparisons.

## Part 4: measurement error bias understood through single world intervention graphs

Thus far, we have repeatedly observed that all biases in causal inference relate to confounding. In Part 1, we examined undirected/uncorrelated measurement error bias and found that measurement bias can arise ‘off the null’ without any confounding (Table 3 *G*_2_). In Part 2, we examined population-restriction bias at the end of a study, finding it to be a variety of undirected uncorrelated measurement error bias (Table 4 *G*_5_). In Part 3, we examined population-restriction bias at baseline; of the five biases considered, only one could be classified as confounding bias.

Throughout this article, we encountered challenges in using causal direct acyclic graphs to represent biases that arise from effect modification. The blue arrows that we use to convey this bias in causal dags might make it appear that bias occurs through action at a distance. That causal directed acyclic graphs are limited in representing such biases should come as no surprise because causal DAGs are designed to clarify confounding bias and not other biases (Hernán & Robins, 2024; Pearl, 2009).

To enrich our understanding of bias from measurement error bias and target population restriction – certain forms of which occur without confounding bias – we turn to Single World Intervention Graphs (SWIGs). These are causal diagrams that allow us to read counterfactual dependencies directly off a graph (Richardson & Robins, 2013a). Similar to causal DAGs, Single World Intervention Graphs (SWIGS) are not purpose-built to evaluate measurement error and restriction biases: they function to factorise conditional probability distributions from assumed causal structures so that investigators may evaluate identifiability conditions – or ‘no unmeasured confounding’ (Bulbulia, 2024c). However, because Single World Intervention Graphs (SWIGS) encode assumptions about the relationships of treatments to the counterfactual outcomes that arise after an intervention is made, they may help us to better understand the causal mechanisms at work when there are measurement error biases. We can demonstrate that there is no ‘action at distance’ in measurement error biases. Furthermore, because target population restriction biases can be approached as measurement error biases, our results extend to target restriction biases as well.

Single World Intervention Graphs (SWIGs) operate by ‘node-splitting’ at each intervention (Bulbulia, 2024c; Richardson & Robins, 2013a), dividing the intervention into a random component and a fixed component. Nodes that follow a fixed intervention are relabelled with the value of the intervention depicted in a SWIG. Importantly only one intervention is represented in any given SWIG (we never observe the joint distribution of more than one intervention at a time). Single World Intervention Templates (SWITs) are ‘graph value functions’ that we may use to generate multiple SWIGs (Richardson & Robins, 2013b). Whether we imagine a single-point treatment or sequential treatments, one reads a SWIG just as one would read a causal DAG, ensuring that there are no backdoor paths linking the random part of the node to the outcome. The deterministic part of a node is fixed, preventing confounding in the counterfactual future from the fixed portion of the node unless an open backdoor path arises before a subsequent intervention such that the subsequent intervention is no longer d-separated from the outcome. Again, although SWIGs, like causal DAGs, are built to evaluate the ‘no unmeasured confounding’ assumption of causal inference by factorising observed joint distributions into conditional and marginal distributions associated with a graph, the explicit representation of counterfactual states in a SWIG makes it easier to understand how bias arises in the absence of confounding, without supposing action at a distance.

### Measurement error in the treatment biases causal contrasts because the treatment reporter is a post-treatment collider

Table 7 *G*_1.1_ presents a single world intervention template from which we may generate two counterfactual states of the world under two distinct interventions 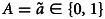. We call the measurement of the intervention a ‘reporter of A’ and denote the state of the reporter under 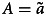 as 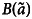. In our convention, if a node in a SWIG (or template) is unobserved, we shade it in grey. *E*^*A*^ denotes an unmeasured variable or set of variables that cause the reporter 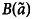 to differ from 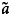, the fixed state of the intervention when *A* is set to 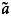. In template Table 7 *G*_1.1_, 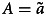 remains unobserved. The only observed nodes are 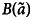 and 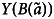, which is the potential outcome for *Y as reported* by 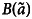. Note that here we include reporters of the unobserved true state of the treatment directly in our representation of the causal order as encoded in our SWIG. Table 7 *G*_1.2_ corresponds to the assumed state of the world when the reporter of *A* is set to *B*(0). Table 7 *G*_1.3_ corresponds to the assumed state of the world when the reporter of *A* is set to *B*(1); in this world, investigators observe *Y*(*B*(1)). We assume that *E*^*A*^ is independent of both *A* and 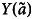. However, we assume that *E*^*A*^ causes 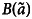 to differ from the true state 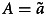. As a result of this misclassification, we have no assurance whether 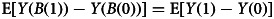. The SWIGs make it apparent that although *A* is independent of *E*^*A*^, 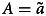 and *E*^*A*^ become statistically entangled in the reporter 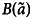, and it is this reporter, not the unobserved true state of 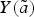, that investigators record.

**Table 7.** Uncorrelated/Undirected Measurement Error in Single-World Intervention Graph. There is no ‘action at a distance’: all measurement errors have causes; errors entering reporters of the treatment and outcome clarify that the treatment reporter induces collider bias, and the outcome reporter induces effect modification during estimation

Measurement Error Bias: Non-directional Uncorrelated Measurement Errors		
**Bias in Treatment Reporter Off-The-Null**		
1.1	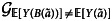	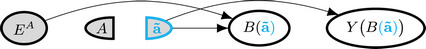
1.2		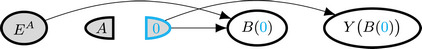
1.3	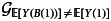	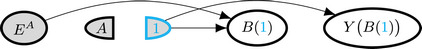
**Bias in Outcome Reporter Off-The-Null**		
2.1	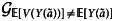	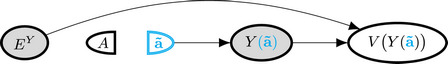
2.2	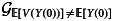	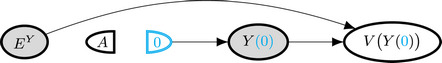
2.3	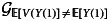	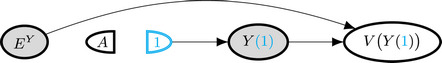
**Measurement Error On The Null: No Bias**		
1.4	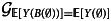	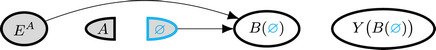
2.4	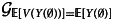	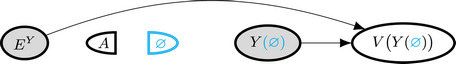

**Key**: 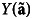 denotes the the true outcome; 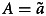 a denotes the true treatment. 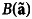 denotes the mismeasured treatment; 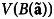 denotes the mismeasured outcome; *E^A^*: unmeasured variable(s) that, together with 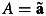 cause measurement: 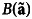; *E^Y^* Unmeasured variable(s) that, together with 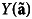 cause measurement: 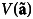; Shaded nodes are unobserved. **Treatment reporter bias**: 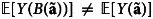; **Outcome reporter bias**: 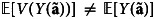.

Consider the following example. Coach Alice randomly assigns one of two running programmes to club runners: *A* = 1 (train), *A* = 0 (do not train). Alice is not interested in estimating the effect of random treatment assignment (the intent-to-treat effect). Rather, she wants to understand the causal effect of training compared with rest – a per-protocol effect. Unknown to Alice, 20% do not follow the programme. Table Table 7 *G*_1.1_ is a SWIG template that presents bias from measurement error in the treatment. The template serves as a ‘graph value function’ that generates SWIGs: Table 7 *G*_1.1_, in which all runners receive *A* = 0 (do not train), and Table 7 *G*_1.2_, in which all runners receive *A* = 1 (train). Here, *B*(0) and *B*(1) denote the reporters of the level of the intervention.

Again, we note that the treatment recorded is not the per-protocol effect 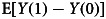 but rather the intention-to-treat effect 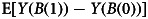. Generally, the effect we obtain will understate the per-protocol effect of training both on the difference scale and the risk ratio scale. Those who were assigned to training but rest will dilute the effect of training: 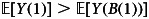. Those who were assigned to rest but who nevertheless train will inflate the expected effect of resting: 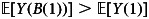. Hence:
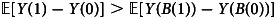
Note that attenuation of a true treatment effect in a setting of uncorrelated errors is not guaranteed (Jurek et al., 2008; Lash et al., 2020). The SWIGs in Table 7 *G*_1.1–1.3_ make the general measurement bias problem clear: although the treatment that is estimated remains d-separated from the potential outcomes, the causal contrast that we obtain at the end of the study is not the treatment we seek and will often (although not always) diminish a true treatment effect because the reporter *under treatment* is a common effect of the unmeasured source of bias and the treatment that has been applied, and it is the outcomes under mismeasured treatments that investigators contrast.

### Measurement error in the outcome biases causal contrasts because the unmeasured error of the outcome is an effect modifier of the outcome reporter

Table 7 *G*_2.1_ presents a single world intervention template from which we may generate two counterfactual states of the world under two distinct interventions 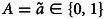. Here, the treatment is observed and recorded without error. Hence we do not include a reporter of the treatment. However, the true outcome is not observed, but only reported with error. *E*^*Y*^ denotes the unmeasured source of error in the reporting of 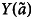, which we assume to be independent of *A* and of *Y*. We shade these nodes in grey because both *E*^*Y*^ and 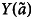 are not observed. The node 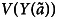 denotes the observed state of *Y* when 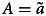. In template Table 7 *G*_2.1_, 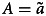 remains unobserved. Table 7 *G*_2.2_ corresponds to the assumed state of the world when *A* = 0 and *Y*(0) is reported with error as *V*(*Y*(0)). Likewise, Table 7 *G*_2.3_ corresponds to the assumed state of the world when *A* = 1 and *Y*(1) is reported with error as *V*(*Y*(1)). We assume that *E*^*Y*^ is independent of *A* and of *Y*. Misclassification will tend to increase the variance of the estimated treatment effect. If the outcome is continuous, the expected difference in the mean of the outcome for the reported outcome may differ from that for the true outcome. How bias affects the outcome will vary depending on the scale we use to evaluate such bias.

Suppose that under training, the athlete runs a marathon in 3 hours, and under rest, they run a marathon in 4 hours. To keep figures easy, we will use round numbers. Suppose the bias in reporting is 1 hour. Thus, we have 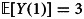, 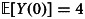; 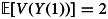 and 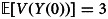.
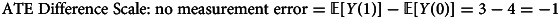


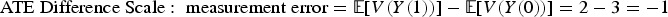

The effect estimates do not differ:
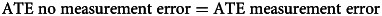
However, consider this bias on the risk ratio scale:
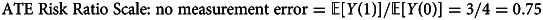

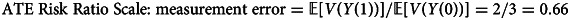
These effect estimates differ:
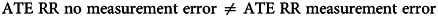
Imagine that the bias was positive, such that runners added an hour to their times – perhaps the runners do not want to stand out. The true risk ratio for the treatment remains 0.75. However, the biased risk ratio for the treatment would become:
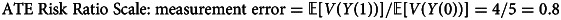
Here we would understate the true treatment effect. The SWIGs (Table 7 *G*_2.1–2.3_) make clear the reason for the scale sensitivity of the bias. Although the source of bias in the outcome (*E*^*Y*^) is independent of the treatment (*A*), *E*^*Y*^ functions as an effect modifier for the reported outcome 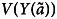.

Consider: it has long been understood that where treatment effects vary across different population strata, an estimate of the causal effect on the risk difference scale will differ from the estimate on the risk ratio scale (Greenland, 2003). Here, we find that reporters of the outcome are subject to similar relativity. For example, we might have constructed a multiplicative error function for the outcome such that we subtract 1 hour if the response is 3 and subtract 1.344 if the response is 4. Under this error function, the risk ratio would remain stable at 0.75 irrespective of whether the outcome was measured with error; however, the risk difference would no longer be constant.

Note that we have encountered SWIGs Table 7 *G*_1.2–1.3_ and Table 7 *G*_2.2–2.3_ before. These causal graphs are structurally equivalent to the causal directed acyclic graph in Part 1 Table 3 *G*_2_, in which we considered uncorrelated independent measurement error. Moreover, Table 7 *G*_2.2–2.3_ is equivalent to Part 2 Table 4 *G*_5_, in which we considered target-restriction bias without confounding; and Part 3 Table 5 *G*_1_, *G*_2_, *G*_4_ in which we considered target population restriction biases in the analytic sample at the start of the study. Single world intervention graphs are useful in providing a more detailed representation of causality in which the biases that give rise to biased causal effect estimates when there is measurement error bias ‘off the null’ – such as when restricted representation of the target population by the analytic sample at the start or end invalidates the causal inferences we seek for the target population.

### Measurement error in the treatment or outcome will not modify a strictly ‘null’ effect

As shown in Table 7 *G*_1.4_ and Table 7 *G*_2.4_, if we assume randomisation into treatments, or equivalently if we assume no unmeasured confounding conditional on perfectly measured covariates, we will not expect a biasing path leading to an association between treatment and outcome. However, a strict ‘null’ effect cannot be assumed. Note that we do not use ‘null’ here in the sense of null hypothesis significance testing, where there is no such thing as a ‘null’ effect. Supplementary materials S5 uses SWIGs to describe correlated and directed measurement error and consider how bias correction may be interpreted mechanistically as interventions on reporters.

### Summary of part 4

We have characterised target-population restriction bias, whether at the start or the end of the study, as formally equivalent to undirected uncorrelated measurement error. Here, SWIGs allow us to apply lessons from the study of effect modification to the analysis of measurement error biases. As shown in *G*_2.1–2.3_, SWIGs greatly clarify how measurement error bias for the outcome arises in the absence of confounding bias: the unmeasured causes of error function as effect modifiers of the outcome reporters, such that causal contrasts will differ on at least one scale of effect ‘off the null’. The mathematical explanation in supplementary materials S3 for threats to external validity from right censoring applies equally to threats from left censoring, as does the simulation in supplementary materials S4.

Note that all of the biases we have considered cannot be evaluated by statistical tests. For example, even if investigators were to obtain satisfactory test statistics for metric, configural and scalar equivalence, they would be unable to diagnose target population restriction biases with these tests. Nor would we be able to diagnose other forms of measurement error biases using statistical tests. Rather, they can only evaluate evidence for bias by first representing the causal structures they *assume* hold in the world, and investigating the implications of each assumption one by one. Likewise, we cannot take the invalidation of standard statistical tests as evidence that similar causal effects underpin sample responses to the interventions of interest. Assumptions alone do not clarify the causal realities that give rise to them. A similar point about the role of assumptions in comparative research is made in Schimmelpfennig et al. (2024).

## Conclusions

In causal inference, we start by clearly defining treatments and outcomes and specifying contrasts for hypothetical interventions on a specific scale (such as the additive scale) across a well-defined target population. We then evaluate the prospects for identifying these causal effect estimates on the *full data* – the entire counterfactual dataset where the population is simultaneously observed under each intervention to be contrasted. Obtaining valid contrasts requires that we consider sources of measurement error, and target population restriction bias (also known as ‘selection bias’) – biases that may arise in the absence of confounding by a common cause of the treatment and outcome. Although causal inference is gaining popularity, there is considerable scope to improve habits of reflecting on the threats to valid inference that measurement error bias and target population-restriction bias present. We have considered how these threats become evident in the comparative human sciences, and furthermore, how target population-restriction biases often take the form of measurement error bias. Although SWIGs were developed to evaluate the conditional exchangeability assumption, they also clarify the diagnosis of structural sources of measurement error bias. We have considered more generally that lacking structural assumptions – that is causal assumptions – statistical tests alone are insufficient for diagnosing the implications of measurement error biases, which may be directed, correlated, both directed and correlated, or undirected and uncorrelated. Whether and how we may correct for measurement-error biases requires structural assumptions that we do not obtain from the data alone, refer to supplementary materials S2 and S5.

Nothing I have said here should detract from the importance of seeking species-level knowledge. We should seek such knowledge. Science should seek generalisations where it can because generalisation is knowledge. In my view, there are also ethical reasons – a great many populations remain understudied. Where there are no scientific reasons for restriction, where a valid target population can be stated for a cross-cultural sample and where costs permit, we should seek wider samples. Here, we have considered that problems of measurement-error bias remain even if adequate samples are taken from broader populations. To put the point metaphorically, before investigators venture into the vast wildernesses of human existence, locally understood gardens of human existence must be cultivated. Because a long shadow of measurement error bias casts its shade over nearly every aspect of the human condition that scientists hope to understand, locally understood gardens of human existence remain largely uncultivated. The good news is that standard workflows for causal inference offer investigators practical guidance. We avoid weird (wrongly estimated inferences owing to inappropriate restriction and distortion) inferences in comparative research in the same way that we avoid *weird* inferences in any research by undertaking the following steps:

1. *State a well-defined intervention* **–** clearly define the treatment or exposure to be evaluated. For which exposures do we hope to infer consequences? Which levels of the exposure shall we compare? Why these levels and not others?
2. *State a well-defined outcome* **–** clearly define the outcome to be evaluated. Which consequences are of interest? Which comparisons will be made? At which time scale following exposure are we interested in evaluating outcomes? At which scale of causal contrast are we interested?
3. *Clarify the target population –* use eligibility criteria to define the population to whom the results are meant to generalise, understanding that causal contrasts may differ for different populations, even in the absence of confounding or measurement error biases (Hernán et al., 2016).
4. *Ensure treatments to be compared satisfy causal consistency –* verify that the treatments correspond to interpretable interventions (Hernán & Robins, 2024). Satisfying the causal consistency assumption is a necesary condition for valid causal inference.
5. *Evaluate whether treatment groups, conditional on measured covariates, are exchangeable –* balancing confounding covariates across treatment levels ensures that differences between groups are ‘ignorable’, or equivalently, are conditionally exchangeable, or equivalently, that all backdoor paths have been closed, or equivalently that the treatment and outcome are d-separated. Ensuring that confounders are balanced in the treatments to be compared is a necessary condition for valid causal inference.
6. *Check if the positivity assumption is satisfied –* confirm that all individuals in the target population have a non-zero probability of receiving each treatment level, given their covariates. Satisfying the positivity assumption is a necesary condition for valid causal inference.
7. *Ensure that the measures relate to the scientific questions at hand –* ensure that the data collected and the measures used directly relate to the research question to hand. As part of this, evaluate structural features of measurement error bias. As we have considered, there are manifold possibilities for measurement error bias to obscure the phenomena under study and bias results. For example target-population-restriction biases manifest as measurement error biases where inferences for the analytic sample population differ from inferences for the target population.
8. *Consider strategies to ensure the analytic sample measured at the end of the study represents the target population –* if the distribution of effect modifiers in the study population at the end of treatment differs from the distribution of effect modifiers in target population, the study will be biased in at least one measure of effect.
9. *Clearly communicate the reasoning, evidence and decision-making that inform steps 1–8* – provide transparent and thorough documentation of how steps 1–8 have been made. This includes stating investigator assumptions, disagreements and decisions. Prepare to conduct and report multiple analyses where causal assumptions are debated or ambiguous (Bulbulia, 2024b).

We have seen that the demands of following this workflow in comparative research are more stringent because measurement error biases must be evaluated at every site to be compared. Correlated and directed structures of measurement error bias can distort treatment effect estimates for the broader target population. More fundamentally, the target population in comparative research may not be easily defined, sampled, or when required by the scientific question, appropriately restricted. Methodologists broadly agree on these points but can easily forget them (as discussed in Ghai et al., 2024). We have shown how workflows for causal inference act as essential preflight checklists for ambitious, effective and safe comparative cultural research. These workflows help propel the human sciences forward without overreaching.

## Supporting information

Bulbulia supplementary material Bulbulia supplementary material

## Supplementary material

To view supplementary material for this article, please visit https://doi.org/10.1017/ehs.2024.33

## Research transparency and reproducibility

No data were used in this manuscript. The data used in supplemental materials S4 were simulated.
