# Supplementary material for: Methods in causal inference. Part 3: measurement error and external validity threats
Source: Evol Hum Sci. 2024 Oct 1;6:e42. doi: 10.1017/ehs.2024.33 (PMC11588564; doi:10.1017/ehs.2024.33)
Supplement: Bulbulia supplementary material [file S2513843X24000331sup001.pdf]

Supplementary files for "Methods in Causal Inference Part 2: Interaction, Mediation, and Time-Varying Treatments"

Joseph A. Bulbulia[1]

[1] Victoria University of Wellington, New Zealand ORCID 0000-0002-5861-2056

2024-06-20

Table of contents

S1. Glossary . . . 2
S2. Generalisability and Transportability . . . 4
S3. A Mathematical Explanation for the Difference in Marginal Effects between Censored and Uncensored Populations . . . 5
S4. R Simulation to Clarify Why The Distribution of Effect Modifiers Matters For Estimating Treatment Effects For A Target Population . . . 7
Lessons . . . 11
S5. Bias Correction as Interventions on Reporters . . . 12
References . . . 15

List of Tables

1 Glossary . . . 3
2 Single World Intervention Graph reveals strategies for redressing measurement error. . . . 12
3 Single World Intervention Graph reveals strategies for redressing measurement error when errors are directed or correlated. . . . 13

S1. Glossary

Table 1: Glossary

Term	Definition
**Acyclic**	No variable can be an ancestor or descendant of itself on a causal graph.
**Adjacent Nodes**	Two nodes connected by an arrow are adjacent.
**Adjustment Set**	Variables conditioned to block all backdoor paths between treatment (A) and outcome (Y).
**Ancestor/Descendants**	Nodes connected by directed edges. All descendants of an ancestor can be reached by directed paths.
**Arrow**	Represents direct causation in a causal diagram, pointing from cause to effect.
**Average Treatment Effect (ATE)**	The difference in expected outcomes between treated and untreated units across a specified population. Synonym for Marginal Effect.
**Backdoor Path**	Path that, if not blocked, may associate the treatment and outcome without causality.
**Causal Contrast**	The difference in expected outcomes under different treatment levels.
**Causal Contrast Scale**	The metric for quantifying causal contrasts, chosen based on outcome type and research question.
**Causal Diagram (Causal DAG)**	A graph representing causal relationships to evaluate an identification problem; must be acyclic and describe all confounding, measured and unmeasured for the target population.
**Causal Estimand**	The causal contrast of interest in a study; specifies the intervention, outcome, contrast scale, and target population; stated before analysis.
**Causal Path**	Asserts a change in the parent node will induce a change in its child.
**Censoring**	the sample population is not representative of the target population at baseline (left censoring) or is no longer representative at the end of study (right censoring).
**Collider/"Immorality"**	A variable where two causal paths meet head-to-head, may induce non-causal associations between its parents.
**Conditional Average Treatment Effect (CATE)**	The treatment effect for specific subgroups, defined by measured characteristics.
**Conditioning**	Adjustment for variables in analysis to distinguish causal effects from associations.
**Confounding**	Treatment and outcome are associated independently of causality or are disassociated despite causality, relative to the causal question.
**Confounder**	A variable or set of variables form part of an ideal identification strategy to reduce or eliminate confounding.
**Counterfactual or Potential outcomes**	Hypothetical outcomes under different treatment conditions to be contrasted, only one may be realised for each observed unit.
**Direct Effect (Natural Direct Effect)**	The difference between potential outcomes when the treatment is applied and the mediator is set to no-treatment versus when neither the treatment nor the mediator is applied.
***d*-separation**	Backdoor paths are blocked, satisfying the assumption of 'no unmeasured confounding'.
**Descendant (Child)**	A node causally influenced by a prior node (Parent). A child is a parent's direct descendant.
**Effect-Measure Modifier/Effect-Modifier**	A variable that affects the magnitude or direction of a causal effect.
**Estimator**	Algorithm to compute a statistical estimand from data.
**External Validity/Target Validity**	The generalisability of study findings to the prespecified target population; assumes internal validity.
**Factorisation**	Decomposing the joint probability distribution of variables into a product of conditional probabilities of each variable given its parents.
**Heterogeneous Treatment Effects**	Variation in treatment effects across subgroups or contexts.
**Identification Problem**	Ensure no unmeasured confounding.
**Incident Exposure Effect**	Causal effect of initiating a new treatment.
**Indirect Effect (Natural Indirect Effect)**	The average difference in potential outcomes when the mediator is at its natural value under treatment versus no treatment.
**Instrumental Variable**	Associated with treatment but affecting the outcome only through the treatment, used for estimating causal effects amidst confounding.
**Intention-to-Treat Effect**	The effect of treatment assignment, what random assignment obtains.
**Internal Validity**	The extent to which causal associations in the study population are accurately identified.
**Inverse Probability of Censoring Weights**	Weights used to adjust for bias due to attrition in longitudinal studies.
**Inverse Probability of Treatment Weights**	Weights that create a pseudo-population to achieve treatment balance across conditions.
**Local Markov Assumption**	assumption that a variable is independent of its non-descendants given its immediate parents in a causal graph.
**Longitudinal Study/Panel Study**	A research design that repeatedly tracks and measures the same units over time.
**Loss-to-follow-up**	Participant attrition.
**Markov Assumption**	assumption that a variable is independent of its non-descendants given its parents in a causal graph
**Marginal Effect**	Synonym for Average Treatment Effect.
**Measurement Error Bias**	Bias introduced when measurements of variables are inaccurately recorded, either through correlated or direct measurement errors, or when uncorrelated errors mask the true effects.
**Mediator**	A variable through which a treatment affects an outcome.
**Modularity Assumption**	Interventions on one set of variables do not directly alter the conditional distribution of other variables, given their direct causes.
**Node**	Represents a variable in a causal diagram, also called "Vertex"
**Observational Study**	Treatment assignment is not controlled by the investigator.
**Parent/Child**	Adjacent nodes connected by a directed path.
**Path**	Nodes are connected by a sequence of edges. Directed paths follow directed edges.
**Per-Protocol Effect**	The causal effect under full-treatment adherence.
**Prevalent Exposure Effect**	Effect of current or ongoing treatments.
**Propensity Score**	The probability of receiving a treatment based on observed characteristics used for confounding adjustment in observational studies.
**Randomised Treatment Assignment**	Chance treatment assignment.
**Randomised Controlled Trial (RCT)**	Uses random treatment assignment to balance confounders across the treatments to be compared.
**Reverse Causation**	Mistaking the effect for the cause in an analysis.
**Sample Weights**	Adjusts sample data to represent the target population in analysis better.
**Selection Bias**	Systematic errors from non-representative study participation or attrition affecting generalisability.
**Sequentially Treatment**	multiple treatments may be fixed our time-varying
**Single World Intervention Graph (SWIG)**	A graph to obtain causal identification under a single counterfactual treatment regime by splitting nodes into random and fixed components, where the fixed inherits edges directed into the node (parents) and the random inherits edges out (children).
**Single World Intervention Template (SWIT)**	A graph-valued function or template generates SWIGs (is not itself a graph).
**Statistical Estimand**	The parameter of interest in a statistical model, not necessarily causal.
**Statistical Estimate**	The value obtained for a statistical estimand from data analysis.
**Statistical Model**	Describes covariance between variables; without structural assumptions, statistical models do not identify causal effects.
**Structural Model**	Assumptions about causal relationships encoded in diagrams, essential for identifying causality from statistical associations.
**Study Population**	The population from which data are collected, also called the "sample population."
**Target Population**	The broader population to which study results are intended to apply.
**Target Trial**	An observational study emulating an ideal experiment by pre-specifying a causal estimand, eligibility criteria, and data ordering for an incident exposure effect.
**Time-Varying Confounding**	Confounding that changes over time, complicating causal effect estimation using standard methods.
**Total Effect**	The difference in mean potential outcomes under contrasted treatments in a study.

S2. Generalisability and Transportability

Generalisability: When a study sample is drawn randomly from the target population, we may generalise from the sample to the target population as follows.

Suppose we sample randomly from the target population, where:

- n_S denotes the size of the study's analytic sample S.
- N_T denotes the total size of the target population T.
- $\widehat{ATE}_{n_S}$ denotes the estimated average treatment effect in the analytic sample S.
- ATE_T denotes the true average treatment effect in the target population T.
- ϵ denotes an arbitrarily small positive value.

Assuming the rest of the causal inference workflow goes to plan (randomisation succeeds, there is no measurement error, no model misspecification, etc.), as the random sample size n_S increases, the estimated treatment effect in the analytic sample S converges in probability to the true treatment effect in the target population T:

$$\lim_{n_S \to N_T} P(|\widehat{ATE}_{n_S} - ATE_T| < \epsilon) = 1$$

for any small positive value of ϵ.

Transportability: When the analytic sample is not drawn from the target population, we cannot directly generalise the findings. However, we can transport the estimated causal effect from the source population to the target population under certain assumptions. This involves adjusting for differences in the distributions of effect modifiers between the two populations. The closer the source population is to the target population, the more plausible the transportability assumptions are, and the less we need to rely on complex adjustment methods. Suppose we have an analytic sample n_S drawn from a source population S, and we want to estimate the average treatment effect in a target population T. Define:

$\widehat{ATE}_S$ as the estimated average treatment effect in the analytic sample drawn from the source population S. $\widehat{ATE}_T$ as the estimated average treatment effect in the target population T. $f(n_S, R)$ as the mapping function that adjusts the estimated effect in the analytic sample using a set of measured covariates R, allowing for valid projection from the source population to the target population.

The transportability assumption is that there exists a function f such that:

$$\widehat{ATE}_T = f(n_S, R)$$

Finding a suitable function f is the central challenge in adjusting for sampling bias and achieving transportability (Bareinboim & Pearl, 2013; Dahabreh et al., 2019; Deffner et al., 2022; Westreich et al., 2017).

S3. A Mathematical Explanation for the Difference in Marginal Effects between Censored and Uncensored Populations

This appendix provides an explanation for why marginal effects may differ between the censored and uncensored sample population in the absence of unmeasured confounding.

Definitions:

- A: Exposure variable, where a represents the reference level and a^* represents the comparison level
- Y: Outcome variable
- F: Effect modifier
- C: Indicator for the uncensored population ($C = 0$) or the censored population ($C = 1$)

Average Treatment Effects:

The average treatment effects for the uncensored and censored populations are defined as:

$$\Delta_{\text{uncensored}} = \mathbb{E}[Y(a^*) - Y(a) \mid C = 0]$$

$$\Delta_{\text{censored}} = \mathbb{E}[Y(a^*) - Y(a) \mid C = 1]$$

Potential Outcomes:

By causal consistency, potential outcomes can be expressed in terms of observed outcomes:

$$\Delta_{\text{uncensored}} = \mathbb{E}[Y \mid A = a^*, C = 0] - \mathbb{E}[Y \mid A = a, C = 0]$$

$$\Delta_{\text{censored}} = \mathbb{E}[Y \mid A = a^*, C = 1] - \mathbb{E}[Y \mid A = a, C = 1]$$

Law of Total Probability:

Applying the Law of Total Probability, we can weight the average treatment effects by the conditional probability of the effect modifier F:

$$\Delta_{\text{uncensored}} = \sum_f \{\mathbb{E}[Y \mid A = a^*, F = f, C = 0] - \mathbb{E}[Y \mid A = a, F = f, C = 0]\} \times \Pr(F = f \mid C = 0)$$

$$\Delta_{\text{censored}} = \sum_f \{\mathbb{E}[Y \mid A = a^*, F = f, C = 1] - \mathbb{E}[Y \mid A = a, F = f, C = 1]\} \times \Pr(F = f \mid C = 1)$$

Assumption of Informative Censoring:

We assume that the distribution of the effect modifier F differs between the censored and uncensored populations:

$$\Pr(F = f \mid C = 0) \neq \Pr(F = f \mid C = 1)$$

Under this assumption, the probability weights used to calculate the marginal effects for the uncensored and censored populations differ.

Effect Estimates for Censored and Uncensored Populations:

Given that $\Pr(F = f \mid C = 0) \neq \Pr(F = f \mid C = 1)$, we cannot guarantee that:

$$\Delta_{\text{uncensored}} = \Delta_{\text{censored}}$$

The equality of marginal effects between the two populations will only hold if there is a universal null effect (i.e., no effect of the exposure on the outcome for any individual) across all units, by chance, or under specific conditions discussed by VanderWeele & Robins (2007) and further elucidated by Suzuki et al. (2013). Otherwise:

$$\Delta_{\text{uncensored}} \neq \Delta_{\text{censored}}$$

Furthermore, VanderWeele (2012) proved that if there is effect modification of A by F, there will be a difference in at least one scale of causal contrast, such that:

$$\Delta^{\text{risk ratio}}_{\text{uncensored}} \neq \Delta^{\text{risk ratio}}_{\text{censored}}$$

or

$$\Delta^{\text{difference}}_{\text{uncensored}} \neq \Delta^{\text{difference}}_{\text{censored}}$$

For comprehensive discussions on sampling and inference, refer to Dahabreh & Hernán (2019) and Dahabreh et al. (2021).

S4. R Simulation to Clarify Why The Distribution of Effect Modifiers Matters For Estimating Treatment Effects For A Target Population

First, we load the `stdReg` library, which obtains marginal effect estimates by simulating counterfactuals under different levels of treatment (Sjölander, 2016). If a treatment is continuous, the levels can be specified.

We also load the `parameters` library, which creates nice tables (Lüdecke et al., 2020).

```
#|label: loadlibs

# to obtain marginal effects
if (!requireNamespace('stdReg', quietly = TRUE)) install.packages('stdReg')
library(stdReg)

#  to view data
if (!requireNamespace('skimr', quietly = TRUE)) install.packages('skimr')
library(skimr)

# to create nice tables
if (!requireNamespace('parameters', quietly = TRUE)) install.packages('parameters')
library(parameters)
```

Next, we write a function to simulate data for the sample and target populations.

We assume the treatment effect is the same in the sample and target populations, that the coefficient for the effect modifier and the coefficient for interaction are the same, that there is no unmeasured confounding throughout the study, and that there is only selective attrition of one effect modifier such that the baseline population differs from the analytic sample population at the end of the study.

That is: **the distribution of effect modifiers is the only respect in which the sample will differ from the target population.**

This function will generate data under a range of scenarios. Refer to documentation in the `margot` package: Bulbulia (2024)

```
# function to generate data for the sample and population,
# Along with precise sample weights for the population, there are differences
# in the distribution of the true effect modifier but no differences in the treatment effect
# or the effect modification. all that differs between the sample and the population is
# the distribution of effect modifiers.

# seed
set.seed(123)

# simulate data -- you can use different parameters
data <- margot::simulate_ate_data_with_weights(
  n_sample = 10000,
  n_population = 100000,
  p_z_sample = 0.1,
  p_z_population = 0.5,
  beta_a = 1,
  beta_z = 2.5,
  noise_sd = 0.5
)
```

```
# inspect
# skimr::skim(data)
```

We have generated both sample and population data.

Next, we verify that the distributions of effect modifiers differ in the sample and in the target population:

```
# obtain the generated data
sample_data <- data$sample_data
population_data <- data$population_data

# check imbalance
table(sample_data$z_sample) # type 1 is rare
```

```

   0    1 
9055  945 
```

```
table(population_data$z_population) # type 1 is common
```

```

    0     1 
49916 50084 
```

The sample and population distributions differ.

Next, consider the question: 'What are the differences in the coefficients that we obtain from the study population at the end of the study, compared with those we would obtain for the target population?'

First, we obtain the regression coefficients for the sample. They are as follows:

```
# model coefficients sample
model_sample  <- glm(y_sample ~ a_sample * z_sample,
  data = sample_data)

# summary
parameters::model_parameters(model_sample, ci_method = 'wald')
```

```
Parameter            | Coefficient |       SE |         95% CI | t(9996) |      p
--------------------------------------------------------------------------------
(Intercept)          |   -6.89e-03 | 7.38e-03 | [-0.02, 0.01]  |   -0.93 | 0.350
a sample             |        1.01 |     0.01 | [ 0.99, 1.03]  |   95.84 | < .001
z sample             |        2.47 |     0.02 | [ 2.43, 2.52]  |  104.09 | < .001
a sample × z sample  |        0.51 |     0.03 | [ 0.44, 0.57]  |   14.82 | < .001
```

Next, we obtain the regression coefficients for the weighted regression of the sample. Notice that the coefficients are virtually the same:

```
# model the sample weighted to the population, again note that these coefficients are similar
model_weighted_sample <- glm(y_sample ~ a_sample * z_sample,
  data = sample_data, weights = weights)

# summary
summary(parameters::model_parameters(model_weighted_sample,
  ci_method = 'wald'))
```

```
Parameter            | Coefficient |         95% CI |      p
-------------------------------------------------------------
(Intercept)          |   -6.89e-03 | [-0.03, 0.01] | 0.480 
a sample             |        1.01 | [ 0.98, 1.04] | < .001
z sample             |        2.47 | [ 2.45, 2.50] | < .001
a sample × z sample  |        0.51 | [ 0.47, 0.55] | < .001

Model: y_sample ~ a_sample * z_sample (10000 Observations)
Residual standard deviation: 0.494 (df = 9996)
```

We might be tempted to infer that weighting wasn't relevant to the analysis. However, we'll see that such an interpretation would be a mistake.

Next, we obtain model coefficients for the population. Note again there is no difference – only narrower errors owing to the large sample size.

```r
# model coefficients population -- note that these coefficients are very similar.
model_population <- glm(y_population ~ a_population * z_population,
  data = population_data)

parameters::model_parameters(model_population, ci_method = 'wald')
```

```
Parameter                   | Coefficient |       SE |         95% CI | t(99996) |      p
-----------------------------------------------------------------------------------------
(Intercept)                 |    2.49e-03 | 3.18e-03 | [ 0.00, 0.01] |     0.78 | 0.434 
a population                |        1.00 | 4.49e-03 | [ 0.99, 1.01] |   222.35 | < .001
z population                |        2.50 | 4.49e-03 | [ 2.49, 2.51] |   556.80 | < .001
a population × z population |        0.50 | 6.35e-03 | [ 0.49, 0.51] |    78.80 | < .001
```

Again, there is no difference. That is, we find that all model coefficients are practically equivalent. The different distribution of effect modifiers does not result in different coefficient values for the treatment effect, the effect-modifier 'effect,' or the interaction of the effect modifier and treatment.

Consider why this is the case: in a large sample where the causal effects are invariant – as we have simulated them to be – we will have good replication in the effect modifiers within the sample, so our statistical model can recover the *coefficients* for the population without challenge.

However, in causal inference, we are interested in the marginal effect of the treatment within a population of interest or within strata of this population. That is, we seek an estimate for the counterfactual contrast in which everyone in a pre-specified population or stratum of a population was subject to one level of treatment compared with a counterfactual condition in which everyone in a population was subject to another level of the same treatment.

The marginal effect estimates will differ in at least one measure of effect when the analytic sample population has a different distribution of effect modifiers compared to the target population.

To see this, we use the `stdReg` package to recover marginal effect estimates, comparing (1) the sample ATE, (2) the true oracle ATE for the population, and (3) the weighted sample ATE. We will use the outputs of the same models above. The only difference is that we will calculate marginal effects from these outputs. We will contrast a difference from an intervention in which everyone receives treatment = 0 with one in which everyone receives treatment = 1; however, this choice is arbitrary, and the general lessons apply irrespective of the estimand.

First, consider this Average Treatment Effect for the analytic population:

```r
# What inference do we draw?
# we cannot say the models are unbiased for the marginal effect estimates.
```

```r
# regression standardisation
library(stdReg) # to obtain marginal effects

# obtain sample ate
fit_std_sample <- stdReg::stdGlm(model_sample,
  data = sample_data, X = 'a_sample')

# summary
summary(fit_std_sample, contrast = 'difference', reference = 0)
```

```
Formula: y_sample ~ a_sample * z_sample
Family: gaussian
Link function: identity
Exposure:  a_sample
Reference level:  a_sample = 0
Contrast:  difference

  Estimate Std. Error lower 0.95 upper 0.95
0     0.00     0.0000       0.00       0.00
1     1.06     0.0101       1.04       1.08
```

The treatment effect is given as a 1.06 unit change in the outcome across the analytic population, with a confidence interval from 1.04 to 1.08.

Next, we obtain the true (oracle) treatment effect for the target population under the same intervention:

```r
## note the population effect is different

# obtain true ate
fit_std_population <- stdReg::stdGlm(model_population,
  data = population_data, X = 'a_population')

# summary
summary(fit_std_population, contrast = 'difference', reference = 0)
```

```
Formula: y_population ~ a_population * z_population
Family: gaussian
Link function: identity
Exposure:  a_population
Reference level:  a_population = 0
Contrast:  difference

  Estimate Std. Error lower 0.95 upper 0.95
0     0.00    0.00000       0.00       0.00
1     1.25    0.00327       1.24       1.26
```

Note that the true treatment effect is a 1.25-unit change in the population, with a confidence bound between 1.24 and 1.26. This is well outside the ATE that we obtain from the analytic population!

Next, consider the ATE in the weighted regression, where the analytic sample was weighted to the target population's true distribution of effect modifiers:

```r
## next try weights adjusted ate where we correctly assign population weights to the sample
fit_std_weighted_sample_weights <- stdReg::stdGlm(model_weighted_sample,
  data = sample_data, X = 'a_sample')

# this gives us the right answer
summary(fit_std_weighted_sample_weights, contrast = 'difference', reference = 0)
```

```
Formula: y_sample ~ a_sample * z_sample
Family: gaussian
Link function: identity
Exposure:  a_sample
Reference level:  a_sample = 0
Contrast:  difference

  Estimate Std. Error lower 0.95 upper 0.95
0     0.00     0.0000       0.00       0.00
1     1.25     0.0172       1.22       1.29
```

We find that we obtain the population-level causal effect estimate with accurate coverage by weighting the sample to the target population. So with appropriate weights, our results generalise from the sample to the target population.

Lessons

- **Regression coefficients do not clarify the problem of sample/target population mismatch** — or selection bias as discussed in this manuscript.
- **Investigators should not rely on regression coefficients alone** when evaluating the biases that arise from sample attrition. This advice applies to both methods that authors use to investigate threats of bias. To implement this advice, authors must first take it themselves.
- **Observed data are generally insufficient for assessing threats**. Observed data do not clarify structural sources of bias, nor do they clarify effect-modification in the full counterfactual data condition where all receive the treatment and all do not receive the treatment (at the same level).
- **To properly assess bias, one needs access to the counterfactual outcome** — what would have happened to the missing participants had they not been lost to follow-up or had they responded? The joint distributions over 'full data' are inherently unobservable (Van Der Laan & Rose, 2011).
- **In simple settings, like the one we just simulated, we can address the gap between the sample and target population using methods such as modelling the censoring (e.g., censoring weighting).** However, we never know what setting we are in or whether it is simple—such modelling must be handled carefully. There is a large and growing epidemiology literature on this topic (see, for example, Li et al. (2023)).

Table 2: Single World Intervention Graph reveals strategies for redressing measurement error.

Correction of Reporters Non-Directional Uncorrelated Measurement Errors

Correction as Intervention on Treatment Reporter

1.1 $\mathscr{G}_{\mathbf{h}\left(E^{A}, B(\tilde{a})\right)}$

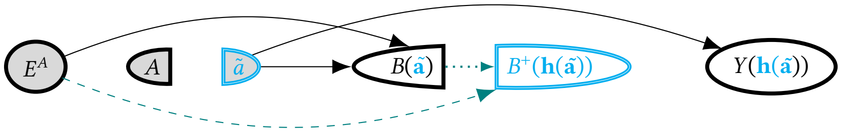

1.2 $\mathscr{G}_{(\tilde{\mathbf{a}}, \mathbf{b})}$

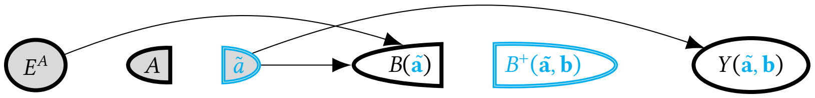

Correction as Intervention on Outcome Reporter

2.1 $\mathscr{G}_{\mathbf{h}\left(E^{Y}, V(\tilde{a})\right)}$

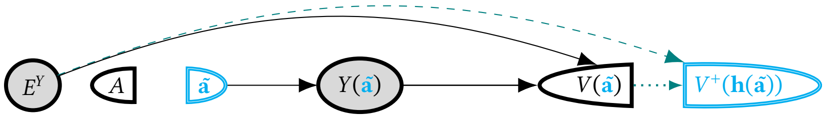

2.2 $\mathscr{G}_{(\tilde{\mathbf{a}}, \mathbf{v})}$

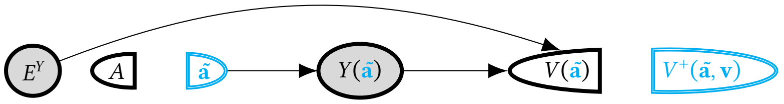

Key: $Y(\mathbf{h}(\cdot))$ denotes the outcome under hidden treatment policy $\mathbf{h}(\cdot)$; A denotes the true treatment, not observed; B denotes the measured treatment; E: cause of the error in the measurement of treatment or outcome or both; ⟶ denotes a causal path; ⟶: bias in a causal path; - -▸ dependencies from time-varying treatments (modified treatment policies) in dynamic Single World Intervention Graphs/Templates. Shaded nodes are unobserved.

S5. Bias Correction as Interventions on Reporters

Single World Intervention Graphs (SWIGs) help us understand why bias correction works. We can think of bias correction without relying on mathematically restrictive models by considering reporters of the true but unobserved states of the world as elements of a causal reality that we represent in SWIGs.

Table 2$\mathscr{G}_{1.1}$ shows how to represent the true counterfactual outcome as a function $Y(\mathbf{h}(E^{A}, B(\tilde{a})))$. If this function were known, we could intervene to correct the bias in reporter B when $A = \tilde{a}$ to obtain $Y(\tilde{a})$. The dotted green arrows indicate the counterfactual variables whose functional relationship to the observed values $B(\tilde{a})$ are relevant for correcting this bias. Like an optometrist fitting spectacles to correct vision, knowing how $B(\tilde{a})$ relates to E^{A} would allow us to recover $A = \tilde{a}$ from $B(\tilde{a})$ and thus obtain $\mathbb{E}[Y(\tilde{a})]$ from $\mathbb{E}[Y(B(\tilde{a}))]$.

Similarly, Table 2$\mathscr{G}_{1.2}$ shows how to represent the true counterfactual outcome as a function $V(\mathbf{h}(E^{Y}, V(\tilde{a})))$. If this function were known, we could intervene to correct the bias of outcome reporter $V(\tilde{a})$ when $A = \tilde{a}$ to recover the true state $Y(\tilde{a})$ from its distorted representation in $V(\tilde{a})$. The dotted green arrows indicate the counterfactual variables relevant for correcting this bias. Knowing how $V(\tilde{a})$ relates to E^{Y} would allow us to recover $Y(\tilde{a})$ from $V(\tilde{a})$.

Table 3$\mathscr{G}_{1.1-1.2}$ reveals that obtaining corrections for biased reporters requires additional information when there is a directed measurement error. In this setting, bias correction requires knowledge of a function in which the treatment and unmeasured sources of error interact to distort reported potential outcomes under treatment. The SWIG shows that directed measurement error bias can occur if the treatment affects the outcome reporter, even without a direct effect of the treatment on the error terms of the outcome reporter.

Table 3: Single World Intervention Graph reveals strategies for redressing measurement error when errors are directed or correlated.

Correction of Reporters Under Directed and Correlated Measurement Errors

Intervention on Reporter to Correct Directed Measurement Error

1.1 $\mathcal{G}_{\mathbf{h}\left((E^Y, \tilde{a}, Y(\tilde{a})\right)}$

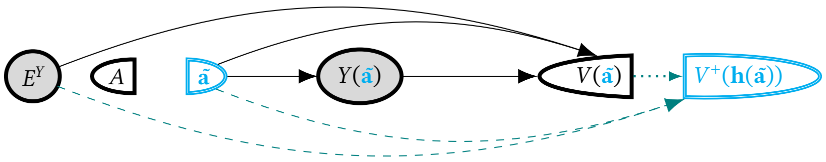

1.2 $\mathcal{G}_{(\tilde{a}, v)}$

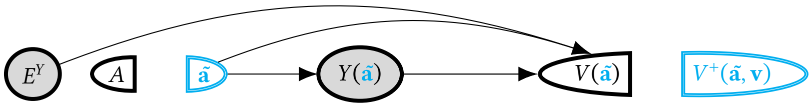

Intervention on Reporter to Correct Correlated Measurement Error

2.1 $\mathcal{G}_{\mathbf{h}\left(E^{AY}, B(\tilde{a}), V(\tilde{a})\right)}$

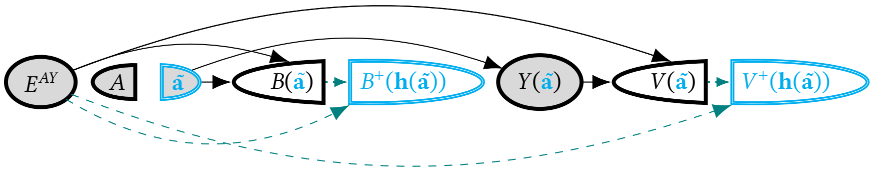

2.2 $\mathcal{G}_{(\tilde{a}, b, v)}$

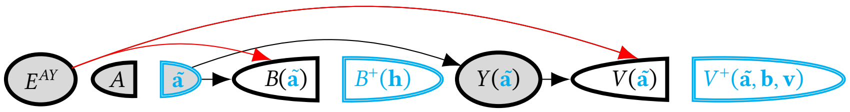

Key: $Y(\mathbf{g}(\cdot))$ denotes the outcome under hidden treatment policy $\mathbf{g}(\cdot)$; A denotes the true treatment, not observed; B denotes the measured treatment; E: cause of the error in the measurement of treatment or outcome or both; ⟶ denotes a causal path; ⟶ (red): bias in a causal path; - - ▸ dependencies from time-varying treatments (modified treatment policies) in dynamic Single World Intervention Graphs/Templates. Shaded nodes are unobserved.

Table 3$\mathscr{G}_{2.1-2.2}$ clarifies that correlated biases in the errors of the treatment and outcome reporters create additional demands for measurement error correction. The behaviour of the correlated error must be evaluated for both $B(\tilde{a})$ and $V(\tilde{a})$. To obtain $V(\tilde{a})$, we must first obtain $\tilde{a}$ from a function $f_B(B(\tilde{a}), E^{AY})$, which cannot be derived from the data because E^{AY} is unobserved. Similarly, a function that recovers $Y(\tilde{a})$ from $V(\tilde{a})$ cannot be obtained from the data because of the unobserved E^{AY}. Further complications arise when considering bias in settings with both directed and correlated measurement errors.

Recall from the main article **Part 3** that we considered how the distribution of effect modifiers across populations complicates inference. These problems are compounded when we include treatment and outcome reporters in our SWIGs. Even if treatment effects were constant across populations, there might be effect modification in the mismeasurement of treatments across populations. Statistical tests alone cannot distinguish between effect modification from treatment effect heterogeneity and effect modification from heterogeneous reporting of treatments or outcomes.

Summary

Our interest in SWIGs has been to understand the causal underpinnings of certain population restriction biases and measurement error biases that arise absent confounding biases. Even assuming strong sequential exchangeability, we can use SWIGs to clarify the mechanisms by which non-confounding biases operate, methods for correcting such biases, and the challenges of comparative research where the distribution of effect modifiers of bias in reporters must be considered to obtain valid causal contrasts for potential outcomes under treatment.

Considerations when using Single World Intervention Graphs for clarifying structural sources of measurement error bias (and other biases):

1. There must be a directed edge from a latent variable to its reporter.
2. If the reporter of the treatment has an arrow entering it from another variable, and causal contrasts are obtained from outcomes under-reported treatments, there will generally be measurement error bias on at least one causal contrast scale (ignoring accidental cancellations of errors), see main article **Part 4**.
3. Likewise, if the reporter of an outcome has an arrow entering it from another variable, and causal contrasts are obtained from reported outcomes, there will generally be measurement error bias on at least one causal contrast scale (ignoring accidental cancellations of errors); see the main article, Part 4.
4. We cannot often control for measurement error biases by *conditioning* on variables in the model because these biases are not confounding biases.
5. However, if the functions that lead to differences between unobserved variables of interest and their reporters are known, investigators can correct for such differences by reweighting the data or applying direct corrections (Carroll et al., 2006; Lash et al., 2009).
6. Certain population restriction biases can be viewed as varieties of measurement error bias, as discussed in the main articles **Part 2** and **Part 3**. SWIGs clarify that certain measurement error biases arise from effect modification, where the error term interacts with the underlying variable of interest, as discussed in the main article **Part 4**.
7. Using SWIGs to approach measurement errors as effect modification is useful because errors might not operate at all intervention levels. Causal DAGs do not readily allow investigators to appreciate these prospects.
8. Despite the formal equivalence of certain forms of measurement error bias and certain forms of population restriction bias, we may use Single World Intervention Graphs to show that both biases may operate together and in conjunction with confounding biases. We would add effect modifier nodes to the SWIGs in Table 2 and Table 3.
9. Despite the utility of Single World Intervention Graphs (and causal DAGs) for clarifying structural features of bias, whether confounding or otherwise, investigators should not be distracted from the goal when using these tools: to understand whether and how valid causal effects may be obtained from observational data for the populations of interest. Every inclination to use causal diagrams should be resisted if their use

complicates this objective.

References

Bareinboim, E., & Pearl, J. (2013). A general algorithm for deciding transportability of experimental results. *Journal of Causal Inference*, *1*(1), 107–134.

Bulbulia, J. A. (2024). *Margot: MARGinal observational treatment-effects*. https://doi.org/10.5281/zenodo.10907724

Carroll, R. J., Ruppert, D., Stefanski, L. A., & Crainiceanu, C. M. (2006). *Measurement error in nonlinear models: A modern perspective*. Chapman; Hall/CRC.

Dahabreh, I. J., Haneuse, S. J. A., Robins, J. M., Robertson, S. E., Buchanan, A. L., Stuart, E. A., & Hernán, M. A. (2021). Study designs for extending causal inferences from a randomized trial to a target population. *American Journal of Epidemiology*, *190*(8), 1632–1642.

Dahabreh, I. J., & Hernán, M. A. (2019). Extending inferences from a randomized trial to a target population. *European Journal of Epidemiology*, *34*(8), 719–722. https://doi.org/10.1007/s10654-019-00533-2

Dahabreh, I. J., Robins, J. M., Haneuse, S. J., & Hernán, M. A. (2019). Generalizing causal inferences from randomized trials: Counterfactual and graphical identification. *arXiv Preprint arXiv:1906.10792*.

Deffner, D., Rohrer, J. M., & McElreath, R. (2022). A Causal Framework for Cross-Cultural Generalizability. *Advances in Methods and Practices in Psychological Science*, *5*(3), 25152459221106366. https://doi.org/10.1177/25152459221106366

Lash, T. L., Fox, M. P., & Fink, A. K. (2009). *Applying quantitative bias analysis to epidemiologic data*. Springer.

Li, W., Miao, W., & Tchetgen Tchetgen, E. (2023). Non-parametric inference about mean functionals of non-ignorable non-response data without identifying the joint distribution. *Journal of the Royal Statistical Society Series B: Statistical Methodology*, *85*(3), 913–935.

Lüdecke, D., Ben-Shachar, M. S., Patil, I., & Makowski, D. (2020). Extracting, computing and exploring the parameters of statistical models using R. *Journal of Open Source Software*, *5*(53), 2445. https://doi.org/10.21105/joss.02445

Sjölander, A. (2016). Regression standardization with the R package stdReg. *European Journal of Epidemiology*, *31*(6), 563–574. https://doi.org/10.1007/s10654-016-0157-3

Suzuki, E., Mitsuhashi, T., Tsuda, T., & Yamamoto, E. (2013). A counterfactual approach to bias and effect modification in terms of response types. *BMC Medical Research Methodology*, *13*(1), 1–17.

Van Der Laan, M. J., & Rose, S. (2011). *Targeted Learning: Causal Inference for Observational and Experimental Data*. Springer. https://link.springer.com/10.1007/978-1-4419-9782-1

VanderWeele, T. J. (2012). Confounding and Effect Modification: Distribution and Measure. *Epidemiologic Methods*, *1*(1), 55–82. https://doi.org/10.1515/2161-962X.1004

VanderWeele, T. J., & Robins, J. M. (2007). Four types of effect modification: a classification based on directed acyclic graphs. *Epidemiology (Cambridge, Mass.)*, *18*(5), 561–568. https://doi.org/10.1097/EDE.0b013e318127181b

Westreich, D., Edwards, J. K., Lesko, C. R., Stuart, E., & Cole, S. R. (2017). Transportability of trial results using inverse odds of sampling weights. *American Journal of Epidemiology*, *186*(8), 1010–1014.
